# Beyond Material Selection: Function-Driven Design of Polymer-Based Microneedles for Transdermal Drug Delivery

**DOI:** 10.3390/jfb17100512

**Published:** 2026-10-11

**Authors:** Teodora Popova, Ivaylo Ganchev, Christina Voycheva

**Affiliations:** 1 Department of Pharmaceutical Technology and Biopharmaceutics, Faculty of Pharmacy, Medical University of Sofia, 1000 Sofia, Bulgaria; 2 Department of Pharmacy and Medical Cosmetics, Burgas State University, 8000 Burgas, Bulgaria; ivaylo-ganchev@uniburgas.bg

**Keywords:** microneedles, transdermal drug delivery, drug release programming, insertion mechanics, polymer fate, translational barriers, biomedical polymers

## Abstract

Polymeric microneedles have emerged as promising platforms for minimally invasive drug delivery, yet their clinical translation remains limited despite extensive proof-of-concept research. This review critically examines polymeric microneedles from a function-driven design perspective, linking classification, fabrication, and material selection to the principal determinants of performance and translational success. Particular attention is given to the interplay among microneedle geometry, polymer properties, skin biomechanics, and application conditions, which collectively define the mechanical window required for reliable insertion. Drug release is considered a programmable design function that should be aligned with dose and pharmacological objectives, while polymer dissolution, degradation, persistence, and potential accumulation are evaluated in relation to safety and regulatory acceptance. Stimuli-responsive and hybrid systems are also assessed by weighing their functional benefits against the additional complexity they introduce. Recurring mechanical, pharmacokinetic, manufacturing, and clinical design–performance mismatches are identified as major contributors to the gap between laboratory success and clinical implementation. Based on these findings, a function-driven framework is proposed that integrates therapeutic objectives, insertion performance, release kinetics, manufacturability, and safety from the earliest stages of development, thereby supporting the rational design of clinically viable next-generation microneedle systems.

## 1. Introduction

The development of alternative routes for drug delivery remains a central objective in pharmaceutical research, driven by persistent limitations of conventional administration pathways. Oral delivery, while convenient, is frequently associated with reduced bioavailability due to gastrointestinal degradation and extensive first-pass metabolism, particularly for peptide- and protein-based therapeutics, as well as stomach irritation and side effects [1,2]. Parenteral administration, although capable of achieving efficient systemic exposure, is often constrained by pain, the need for trained personnel, and reduced patient compliance, especially in the context of chronic therapy [1]. These limitations have stimulated growing interest in minimally invasive delivery strategies that aim to combine the efficacy of injections with the convenience of non-invasive approaches.

Transdermal and intradermal delivery systems have emerged as attractive alternatives, enabling controlled drug release, avoidance of first-pass metabolism, and improved patient adherence [3]. However, the effectiveness of conventional transdermal systems is inherently limited by the barrier function of the stratum corneum, which restricts permeation primarily to small, lipophilic molecules [4]. Consequently, expanding the range of therapeutics suitable for transdermal delivery remains a major challenge in the field.

Microneedle-based systems have been developed to overcome this limitation by creating micron-scale pathways across the stratum corneum, thereby enabling direct access to the viable epidermis and dermis while avoiding nerve-rich regions associated with pain [1,5]. Since their initial conceptualization, microneedles have evolved into a highly versatile platform encompassing multiple delivery strategies, including solid, coated, dissolving, hydrogel-forming, and hollow systems, each tailored to achieve specific pharmacokinetic and therapeutic objectives [5,6]. This versatility has established microneedles as a promising alternative route of administration capable of delivering a wide range of molecules, from small drugs to vaccines, peptides, and nucleic acids [6,7,8].

Among the various microneedle platforms, systems based on polymers have attracted particular attention due to their versatility and tunability.

Polymeric microneedles (MNs) are minimally invasive transdermal drug delivery systems consisting of micron-scale projections fabricated from biodegradable, biocompatible, or water-soluble polymers, which penetrate the stratum corneum to create transient pathways for drug transport into the skin. Their polymeric composition provides considerable flexibility in controlling mechanical strength, dissolution or degradation behavior, swelling, drug loading, and release kinetics profiles [9]. Polymers such as poly(vinylpyrrolidone) (PVP), poly(vinyl alcohol) (PVA), poly(lactic-co-glycolic acid) (PLGA), and polycaprolactone (PCL) have been widely investigated for microneedle fabrication, supporting applications ranging from rapid drug delivery to sustained and long-acting release systems [9,10]. Based on their behavior following skin insertion, dissolving and hydrogel-forming microneedles represent two important classes of polymeric MNs, offering distinct mechanisms for drug release and delivery. This versatility has driven substantial innovation in the field, enabling the development of polymeric microneedles tailored to diverse therapeutic requirements.

Despite the rapid expansion of research activity in this area, a significant gap persists between proof-of-concept studies and clinically translated microneedle systems. Although hundreds of studies report promising in vitro and preclinical results, only a limited number of microneedle-based products have progressed toward commercialization or clinical adoption [10,11]. This discrepancy reflects a fundamental limitation of the current literature: most studies emphasize feasibility rather than critically evaluating whether a given system is optimally designed for its intended therapeutic purpose.

Recent reviews have made important contributions by summarizing advances in polymeric microneedles, manufacturing approaches, and the barriers limiting clinical translation [5,10]. In particular, Moawad et al. (2025) comprehensively discuss the principal limitations and translational challenges that hinder the clinical implementation of microneedle technologies [12]. However, these challenges are largely presented as downstream obstacles rather than as interconnected consequences of design decisions made during system development. Increasing evidence indicates that translational success depends not solely on material properties, but on the alignment between microneedle design, drug release kinetics, and pharmacological requirements [13].

The present review adopts a different organizing perspective. Instead of focusing primarily on material categories or translational limitations in isolation, it examines how geometry, polymer properties, fabrication strategy, skin biomechanics, application conditions, release programming, and polymer fate collectively determine functional performance and ultimately influence translational success. By treating clinical translation as the outcome of design–performance alignment rather than a separate final-stage challenge, this review provides an integrated function-driven framework that connects therapeutic objectives with the key design decisions required for clinically viable polymeric microneedle systems.

Therefore, this review provides a comprehensive analysis of polymeric microneedles as an alternative route for drug delivery, integrating both classical and function-oriented perspectives. It is structured in two complementary parts. The first part outlines microneedle classification, fabrication methods, and material selection, thereby defining the fundamental design space of these systems. The second part adopts a function-driven perspective, focusing on the key determinants of microneedle performance, including mechanical integrity, release programming, stimuli-responsiveness, and polymer fate in the skin, as well as the factors limiting clinical translation. By identifying recurring design–performance relationships, this review aims to establish a rational function-driven framework for the development of next-generation microneedle systems with improved translational potential. Unlike recent reviews that primarily catalog materials, fabrication technologies, or translational barriers, the proposed framework explicitly links therapeutic objectives with the interconnected design parameters that govern insertion performance, drug release, manufacturability, safety, and clinical implementation.

## 2. Classification of Microneedles

Microneedle systems can be broadly classified according to their mechanism of action and mode of drug delivery as solid, coated, hollow, dissolving, biodegradable, implantable and hydrogel-forming microneedles. This classification not only provides a structural overview of the field but also highlights fundamental differences in drug delivery efficiency, dosing control, and clinical applicability [5,11].

The principal microneedle types differ not only in their mechanism of drug delivery but also in the classes of therapeutics for which they are most commonly employed. Solid microneedles primarily create transient microchannels that enhance the subsequent delivery of molecules such as insulin, lidocaine, and model permeants including calcein [14,15]. Coated microneedles carry the active ingredient directly on the needle surface and have been extensively investigated for influenza vaccines and ovalbumin [16,17]. Hollow microneedles function as miniature injection devices capable of delivering liquid formulations, including lidocaine, insulin, influenza vaccines, and monoclonal antibodies [18,19,20,21]. Dissolving microneedles incorporate the drug within a water-soluble polymer matrix, with representative examples including insulin, methotrexate, naltrexone hydrochloride, and influenza vaccines [22,23,24,25]. Biodegradable or implantable microneedles have been investigated for long-acting delivery of levonorgestrel, cabotegravir, and PLGA-based depot formulations [26,27]. In contrast, hydrogel-forming microneedles create swollen polymer networks that enable sustained diffusion of drugs from an attached reservoir and have been studied for insulin, metformin, and macromolecular model compounds such as FITC-dextran [28,29,30].

Below is a comparative table (Table 1) that reflects the general trends reported for microneedles in the literature [1,5,6,7,31,32,33,34,35,36,37,38,39,40,41,42,43,44,45,46,47,48]. Because performance depends on material composition, geometry, drug type, and application conditions, the rankings should be interpreted as relative qualitative estimates rather than absolute values. The qualitative ranking (very low to very high) reflects relative differences among the various microneedle types and does not represent a comparison with alternative drug delivery platforms.

**Figure 1:**
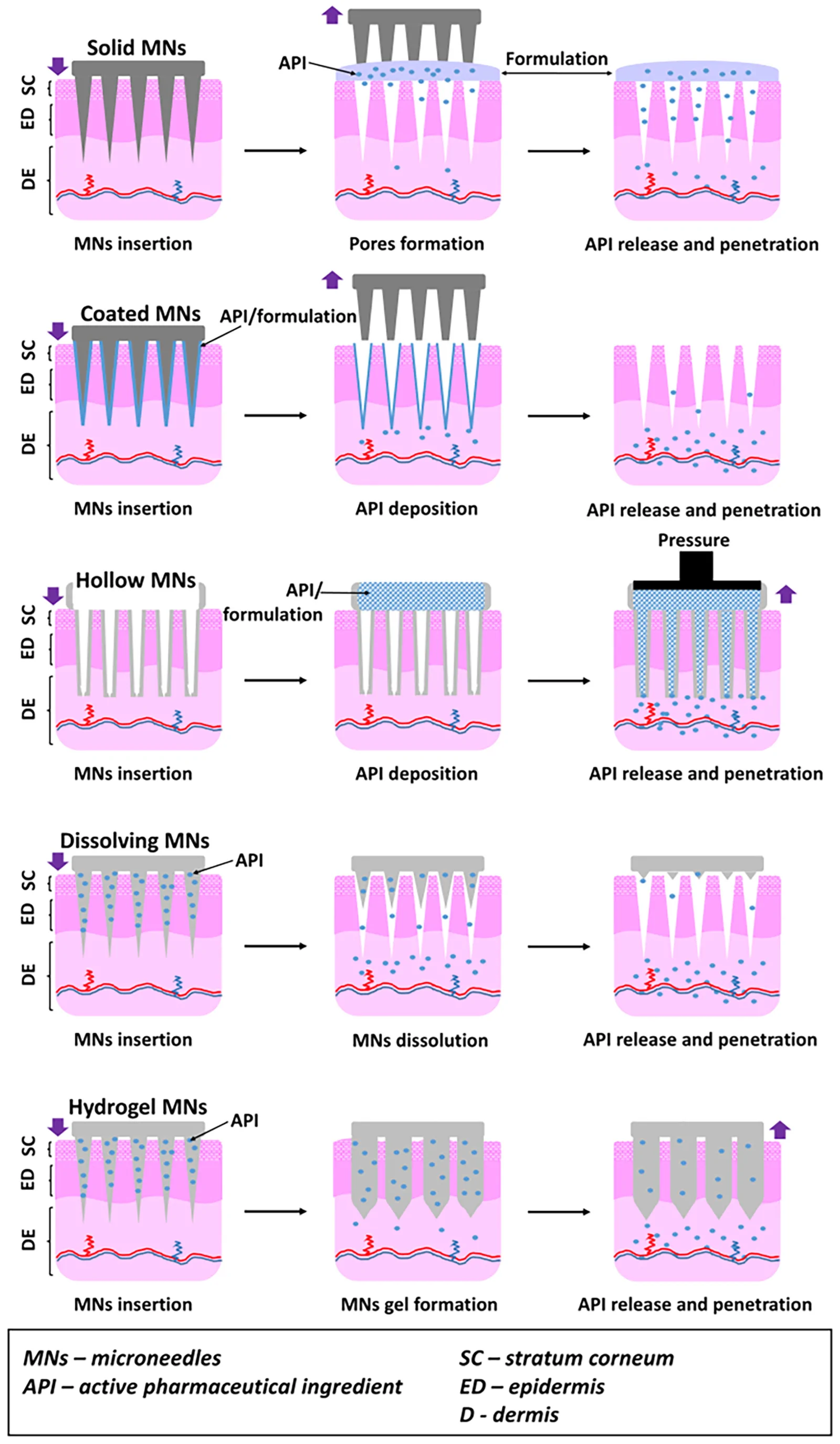
Mechanism-based classification of microneedle systems. The schematic highlights distinct drug delivery strategies, including microchannel formation, surface coating, polymer dissolution, depot formation, diffusion through swollen matrices, and direct liquid infusion.

The classification of microneedle systems based on their mechanism of action is schematically illustrated in Figure 1.

Although these categories are traditionally presented as discrete, many contemporary microneedle systems incorporate hybrid features that combine multiple mechanisms of action. Notably, classification based solely on structure or material does not fully explain the clinical performance of microneedle systems. As will be discussed in subsequent sections, the clinical translation ultimately requires the alignment between design parameters and therapeutic objectives rather than categorical classification alone.

## 3. Fabrication Methods

Microneedle fabrication methods play a critical role in determining the mechanical properties, drug loading capacity, reproducibility, and scalability of polymeric microneedle systems. While a wide range of microfabrication techniques has been explored, the choice of fabrication method is not merely a technical consideration but a fundamental design parameter that directly influences the translational feasibility of the final system [57,58].

To provide a structured comparison of the principal fabrication techniques used in polymeric microneedle development, their key advantages and limitations are summarized in Table 2. As shown, fabrication method selection has direct implications not only for microneedle geometry and drug loading but also for scalability, reproducibility, and overall translational feasibility [57,59,60,61,62].

Hybrid fabrication strategies overcome limitations of individual manufacturing techniques but increase process complexity and quality-control requirements. Consequently, fabrication should be considered a design variable because it directly determines whether critical quality attributes—including geometry, drug distribution, and mechanical reproducibility—can be consistently achieved during scale-up [53].

As indicated in Table 2, no single fabrication method simultaneously satisfies all the requirements for ideal microneedle production, including mechanical robustness, precise drug loading, scalability, and cost-effectiveness. Instead, each technique involves inherent trade-offs that must be carefully balanced according to the intended application.

Micromolding remains the dominant approach due to its versatility and compatibility with drug-loaded systems; however, its sensitivity to processing conditions introduces variability that may hinder reproducibility at scale. In contrast, industrial techniques such as injection molding offer superior consistency and scalability but are limited by material constraints and incompatibility with thermolabile drugs. Emerging technologies such as 3D printing offer unprecedented design flexibility but currently face limitations in resolution and material availability, which restrict their widespread adoption.

Layer-by-layer and multilayer fabrication strategies enable spatial drug compartmentalization and independent control of needle-tip and backing-layer composition, but these additional processing steps increase manufacturing complexity and place greater demands on process reproducibility and quality control [64].

Notably, many of the translational challenges discussed in later sections, particularly those related to dose variability, mechanical failure, and manufacturing reproducibility, can be traced directly to limitations inherent in the selected fabrication method. This observation underscores that fabrication strategy should not be considered a purely technical choice, but rather an integral component of function-driven microneedle design.

In parallel with fabrication strategy, material selection represents a critical determinant of microneedle performance. The physicochemical properties of the polymers used not only influence manufacturability but also define mechanical behavior, drug loading capacity, and release characteristics.

## 4. Materials for Polymeric Microneedles

Microneedle performance is fundamentally governed by the physicochemical properties of the materials used for their fabrication. Polymer selection determines not only the mechanical strength required for effective skin penetration but also drug loading capacity, release kinetics, biocompatibility, and, ultimately, the translational potential of the system. Therefore, materials should not be viewed as passive carriers but as active determinants of microneedle function and clinical applicability [6,13]. Based on their behavior following skin insertion, the polymers used for MN fabrication can be broadly categorized as water-soluble, biodegradable, or hydrogel-forming materials, each offering distinct advantages and limitations (Table 3).

Recent advances in polymer science have enabled the development of stimuli-responsive materials, including thermoresponsive polymers such as poly(N-isopropylacrylamide) (PNIPAm), pH-responsive polymers, and hybrid systems incorporating nanoparticles or bioactive components [10]. These materials offer the potential for on-demand drug release in response to physiological or external stimuli. However, their integration into microneedle systems introduces additional complexity in formulation, fabrication, and regulatory evaluation. In many cases, this added functionality must be carefully justified against the increased risk of variability, reduced reproducibility, and potential safety concerns associated with multi-component systems [10,67].

Primary experimental studies across PVP, PVA, HA, CMC, PCL, PLGA, and PEG-based hydrogel systems collectively demonstrate that similar functional objectives—including reliable insertion, controlled dissolution, sustained release, and mechanical robustness—can be achieved through different polymer families when formulation parameters are appropriately optimized, reinforcing that polymer selection should be guided by functional requirements rather than by a single preferred material class.

An important recent development in polymeric microneedle fabrication is the growing use of photocurable polymers, which enable precise spatial and temporal control over network formation through light-induced crosslinking [68]. Materials such as gelatin methacryloyl (GelMA), polyethylene glycol diacrylate (PEGDA), and methacrylated hyaluronic acid have emerged as versatile platforms because their crosslinking density can be systematically tuned by adjusting parameters including polymer concentration, degree of substitution, photoinitiator content, and light exposure time. Unlike conventionally dried polymer matrices, these systems allow direct modulation of mechanical modulus, swelling behavior, degradation kinetics, and drug diffusion within the polymer network, thereby providing a powerful means of optimizing insertion performance and release programming simultaneously. Experimental studies have shown that GelMA crosslinking conditions can substantially affect microneedle mechanical properties, swelling, degradation behavior, and drug release [69]. In PEGDA-based systems, molecular weight, photoinitiator characteristics, and UV exposure conditions have likewise been shown experimentally to influence microneedle geometry and mechanical stability [70]. Moreover, stereolithography (SLA), digital light processing (DLP), and continuous liquid interface production (CLIP) further extend these capabilities by combining photocurable materials with high-resolution additive manufacturing, enabling precise control over needle geometry, internal microstructures, and mechanical performance [64]. However, translation of light-induced crosslinking to larger-scale manufacturing requires tight control of irradiation conditions, as variations in light exposure, photoinitiator distribution, and curing depth may affect crosslinking uniformity and, consequently, microneedle geometry and mechanical performance. These requirements may complicate process scale-up and reproducibility, particularly when photocuring is integrated with high-resolution additive manufacturing [64,70]. Collectively, these advances illustrate how photopolymerization transforms fabrication from a passive manufacturing step into an active design variable that can be used to engineer clinically relevant microneedle properties.

Material selection is also intrinsically linked to fabrication methods and performance characteristics. Mechanical properties, degradation behavior, and drug–polymer interactions collectively determine whether a given microneedle system can achieve its intended therapeutic function. As will be discussed in later sections, successful translation depends not on the intrinsic novelty of the material but on its alignment with clearly defined pharmacological and clinical requirements.

Across all material classes, a recurring theme is that no polymer is intrinsically optimal for microneedle fabrication. Instead, material suitability is context-dependent and must be evaluated in relation to the intended therapeutic application, required release profile, and clinical use conditions. These findings indicate the central premise of this review: material selection should be guided by functional requirements rather than by material availability or novelty. Integrating material properties with mechanical design, fabrication strategy, and pharmacological objectives represents a critical step toward the rational development of clinically viable microneedle systems.

## 5. Factors Limiting Drug Delivery Efficiency in Polymeric Microneedles and Design Strategies to Overcome Them

**Figure 2:**
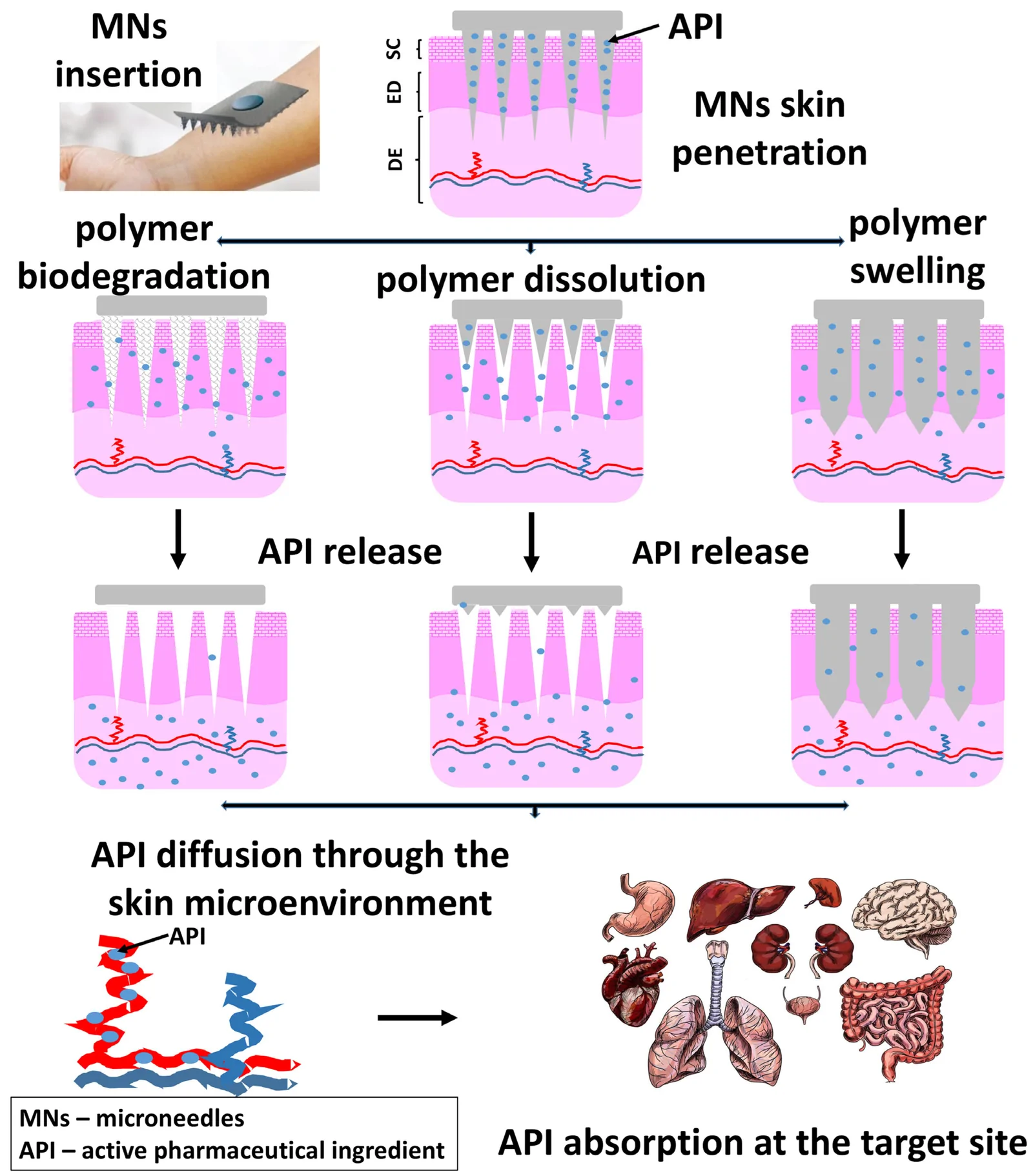
Schematic representation of the drug delivery process from polymeric microneedles and the three principal polymer-behavior mechanisms following skin insertion. Drug delivery involves sequential stages of skin penetration, polymer dissolution, biodegradation, or hydrogel swelling, followed by drug release from the polymeric matrix, diffusion through the skin microenvironment, and subsequent absorption at the target site.

As presented in Figure 2, drug delivery efficiency is governed by a series of interconnected processes and its efficiency is determined by multiple interacting factors rather than by any single design parameter. Mechanical failure during insertion may prevent adequate skin penetration, while inappropriate polymer properties can impair dissolution, swelling, or drug diffusion. Drug-specific factors such as poor solubility, instability, or unfavorable interactions with the polymer matrix may further limit drug release, whereas variations in skin physiology and user-dependent application conditions contribute to delivery variability. Understanding how these interconnected factors influence insertion efficiency, drug loading, release behavior, and skin permeation is therefore essential for the rational design of polymeric microneedles with improved therapeutic performance and greater translational potential [5,6,7,11,13,71].

However, the translational gap in polymeric microneedles is not simply the consequence of insufficient technological maturity but of recurring design–performance mismatches demonstrated across primary experimental studies. For example, mechanically robust microneedles may still fail to penetrate when tip geometry is suboptimal, highly loaded formulations may deliver only a fraction of the incorporated dose because of incomplete dissolution or diffusion limitations, and fabrication-induced variability can compromise dose reproducibility during scale-up. These observations indicate that successful drug delivery depends on coordinated optimization of insertion mechanics, release behavior, manufacturing reproducibility, and clinical usability rather than on any single design parameter [72].

From a systems-level perspective, translational failure in polymeric microneedles can be broadly categorized into four interrelated domains: (i) mechanical–insertion mismatch, (ii) pharmacokinetic–dose mismatch, (iii) manufacturing and scalability limitations, and (iv) clinical–context misalignment. These domains are not independent; instead, they frequently interact, creating compounded barriers that prevent otherwise promising systems from advancing toward clinical implementation. Table 4 summarizes the principal subdomains within these four domains and illustrates their interconnected contributions to translational success or failure.

The conceptual framework presented in Table 4 is further illustrated by representative primary experimental studies (Table S1 [12,64,76,77]) demonstrating how specific design–performance mismatches have been addressed through targeted formulation, geometric, manufacturing, and application-related modifications, ultimately leading to practical design lessons for future microneedle development.

Collectively, these studies demonstrate that successful microneedle optimization rarely results from improving a single parameter in isolation. Instead, experimentally validated solutions consistently emerge from addressing specific design–performance mismatches, whether through geometric refinement, optimization of application conditions, improved manufacturing reproducibility, or incorporation of user-centered design features. These recurring patterns reinforce the function-driven concept developed throughout this review and illustrate how primary experimental evidence can be translated into practical design decisions.

### 5.1. Mechanical–Insertion Performance

Mechanical performance represents the first and indispensable functional requirement for polymeric microneedles intended as an alternative route for drug delivery. Independent of polymer chemistry or drug loading strategy, microneedles must reliably penetrate the stratum corneum without fracturing, bending, or undergoing uncontrolled deformation. Mechanical–insertion performance represents the ability of polymeric microneedles to reliably penetrate the skin while maintaining their structural integrity and creating reproducible microchannels for effective drug delivery. It encompasses the interplay between microneedle geometry, polymer mechanical properties, skin characteristics, and application parameters, including insertion force, angle, method, and residence time, all of which collectively determine insertion efficiency and subsequent drug delivery performance. Failure to meet this requirement compromises drug delivery efficiency, reproducibility, and safety, ultimately limiting clinical translation [5,71,78].

#### 5.1.1. Microneedle Geometry

**Figure 3:**
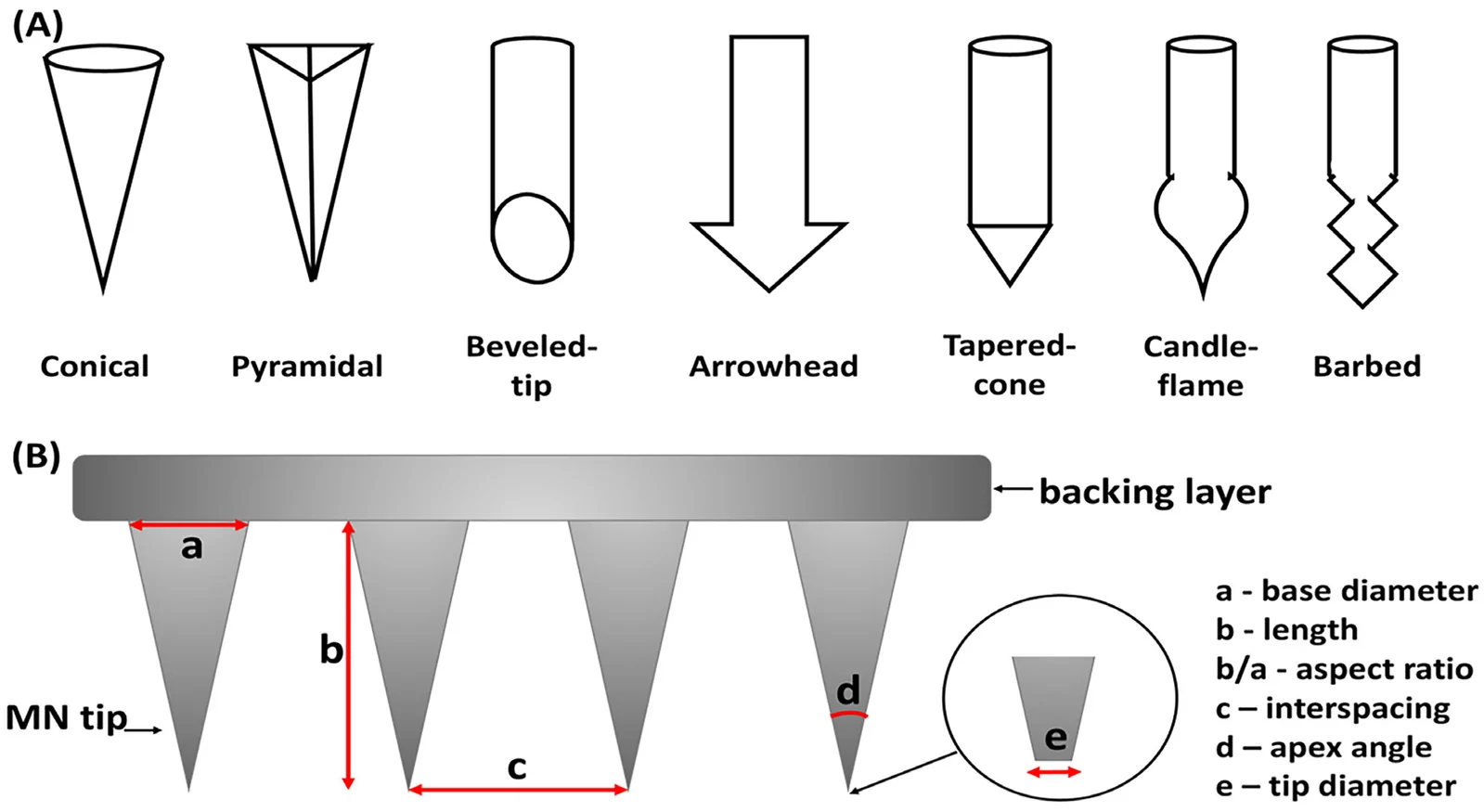
The most common geometrical shapes for microneedles (**A**) and a schematic side view of polymeric microneedles (**B**).

Microneedle geometry plays a critical role in determining overall device performance, as it directly influences mechanical strength, skin insertion efficiency, and drug delivery outcomes. The most commonly investigated geometrical parameters include needle shape, base diameter, needle length, tip diameter, and inter-needle spacing, all of which must be carefully optimized to achieve effective transdermal penetration while maintaining structural integrity. In addition, the aspect ratio and apex angle are key design determinants that govern the balance between insertion capability and mechanical robustness. Taken together, these geometric features, presented in Figure 3, define the interaction of microneedles with the skin barrier and are therefore essential considerations in the rational design of polymeric microneedle systems.

The geometry of microneedle tips strongly influences insertion efficiency, mechanical strength, and pain perception [79,80]. Common shapes include conical, pyramidal, bevelled, and arrowhead structures as visualized in Figure 3A. Among these, conical and pyramidal geometries are most widely used for polymeric microneedles, as they provide a good balance between manufacturability and mechanical stability [81]. Sharp-tipped geometries are preferred because they reduce insertion force and improve skin penetration reliability. In general, sharper and more gradually tapered structures enhance insertion success while minimizing tissue deformation. The influence of microneedle shape on the mechanical performance, insertion behavior, and drug delivery characteristics of polymeric microneedle systems is summarized in Table 5.

Microneedle base diameter

The base diameter determines the mechanical robustness of the microneedle. Larger base diameters increase mechanical strength but may reduce insertion efficiency due to increased skin deformation and higher insertion force requirements. For polymeric microneedles, typical base diameters are in the range of 100–300 µm, with optimization aimed at ensuring sufficient mechanical strength while maintaining a small footprint to reduce the “bed-of-nails” effect in dense arrays [12,71,74,87,88,89,90].

Microneedle length

Needle length is a critical parameter controlling the depth of skin penetration and thus drug delivery location (epidermis vs. dermis). Most effective microneedles are designed to penetrate the stratum corneum and reach the viable epidermis or upper dermis without stimulating deeper nerves or blood vessels. Optimal lengths are typically 300–1000 µm for polymeric microneedles. Shorter needles failed to overcome skin deformation and may lead to incomplete barrier bypass, while excessively long needles increase pain risk and fracture probability [71,88].

The technological requirements for an efficient micro-molding process should be taken into consideration as well, because it is stated that MNs more than 1 mm in length are difficult to remove from the mold [12,71,74,87,88,89,90,91].

Microneedle tip aspect ratio

The aspect ratio (height/base diameter) governs both mechanical strength and insertion capability. High aspect ratios improve penetration ability but increase the risk of buckling and fracture, particularly in polymeric systems. For polymeric microneedles, a commonly reported optimal aspect ratio is 2:1 to 5:1. Within this range, microneedles can maintain structural integrity while still achieving effective skin penetration [12,71,74,87,88,89,90].

Microneedle tip diameter

The tip diameter is one of the most important determinants of insertion force. Sharper tips (smaller diameters) significantly reduce penetration resistance and improve reproducibility of insertion. For efficient skin piercing, tip diameters are generally designed to be below approximately 30 µm, with the smallest feasible tip diameter preferred to minimize insertion force while maintaining structural integrity [12,71,74,87,88,89,90]. MN tips less than 5 µm are easily prone to breaking and bending [12].

Apex (tip) angle

The apex angle defines the sharpness of the microneedle tip and strongly influences insertion force and mechanical stress distribution. A smaller apex angle results in sharper tips and lower insertion force but may reduce mechanical strength. Conversely, larger angles increase robustness but require higher force for insertion. Typical optimized apex angles are in the range of 20–60°, with sharper angles generally preferred for dissolving and hydrogel-forming polymeric microneedles where insertion efficiency is critical [12,71,74,87,88,89,90].

MN tip spacing (interspacing)

Inter-needle spacing is a critical design parameter that influences both insertion efficiency and drug delivery performance. Insufficient spacing may promote the bed-of-nails effect, leading to skin deformation and incomplete insertion, whereas excessive spacing reduces needle density and the total drug-loading capacity of the microneedle array. Typical spacing values range from 300–800 µm, depending on needle height and array density. Optimized spacing ensures adequate skin recovery between insertion points while maintaining sufficient delivery surface area [12,43,50,71,82,88].

Throughout this review, qualitative descriptors describing mechanical and insertion performance are used in accordance with representative quantitative ranges commonly reported in the literature (Supplementary Table S1), recognizing that these values depend on polymer properties, geometry, fabrication conditions, and testing methodology.

Importantly, the suitability of these dimensional ranges depends strongly on the mechanical characteristics of the selected polymer matrix. Elastic modulus, crystallinity, glass transition temperature, molecular weight, and crosslink density collectively determine how a given geometry responds to compressive loading during skin insertion. For example, polymers with higher elastic modulus or greater crystallinity may maintain structural integrity at higher aspect ratios, whereas softer or lower-modulus materials often require larger base diameters, reduced needle lengths, or modified tip geometries to avoid buckling or excessive deformation. Likewise, polymers with glass transition temperatures close to physiological conditions may exhibit altered mechanical behavior during application, further influencing the relationship between geometry and insertion performance. Consequently, geometric parameters should be optimized together with material properties, as identical needle dimensions can produce substantially different insertion efficiency, fracture resistance, and drug-delivery performance depending on the polymer matrix employed [12].

**Figure 4:**
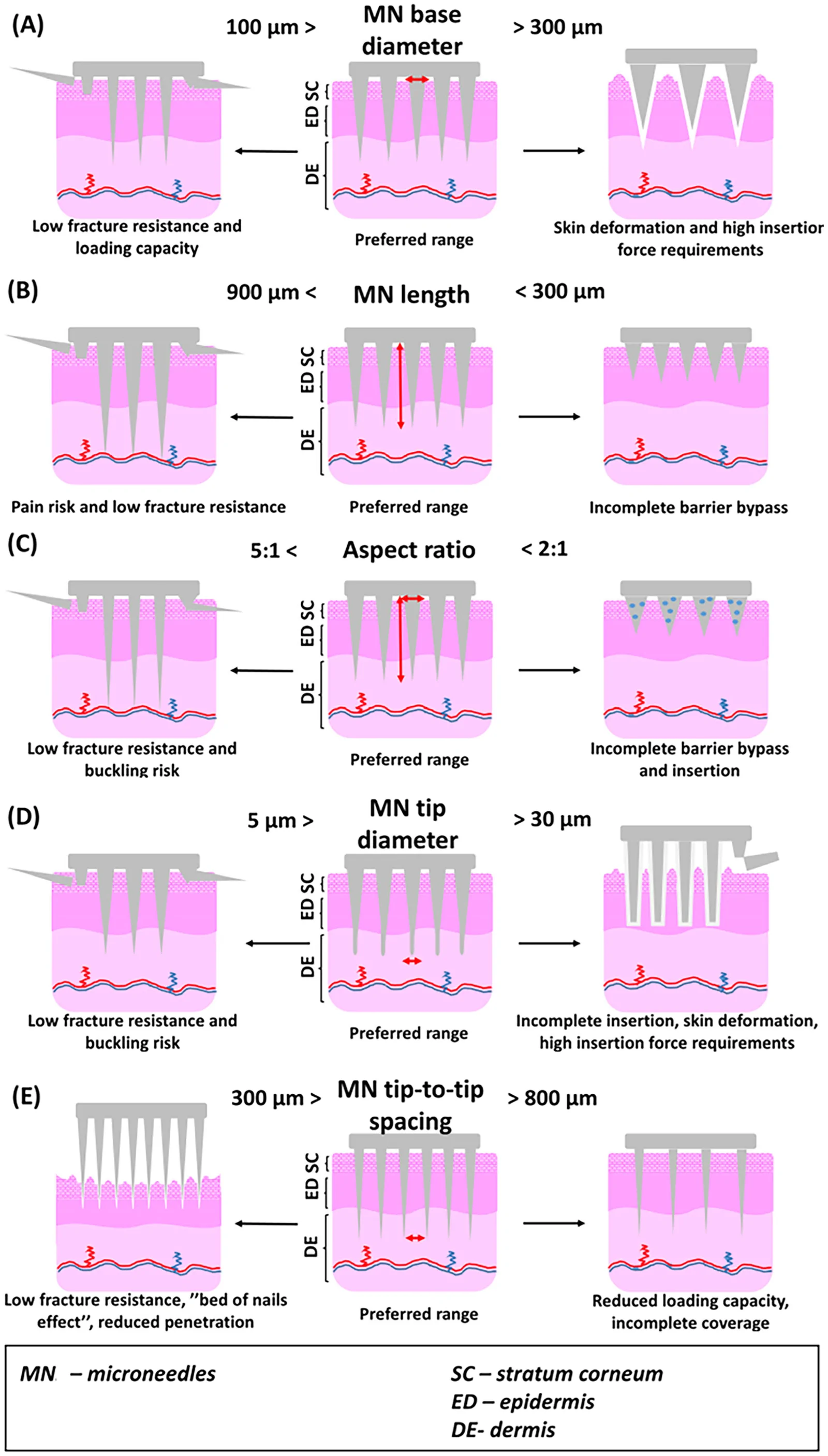
Influence of microneedle geometry on mechanical performance, skin penetration, and drug delivery efficiency. Schematic representation of the effects of MN base diameter (**A**), MN length (**B**), aspect ratio (**C**), MN tip diameter (**D**), and MN tip-to-tip spacing (**E**) on insertion behavior, mechanical stability, and drug delivery performance. The reported geometric ranges are indicative rather than universally applicable, as optimal MN dimensions depend on the material and geometry of the microneedles, anatomical site, application method and force, and the intended depth of skin penetration.

The interplay between microneedle geometry and mechanical performance is summarized in Table 6 and visualized in Figure 4.

Overall, the geometric design of microneedles requires a careful balance between several interdependent performance criteria, including mechanical strength, insertion efficiency, and patient safety. Needle height, base diameter, tip radius, tip angle, and aspect ratio determine stress distribution during insertion and resistance to buckling or fracture [32,93] and must be optimized collectively rather than independently, as improving one feature often compromises another. For example, sharper and higher aspect ratio structures can significantly enhance skin penetration and reduce insertion force, but may also increase the risk of mechanical failure such as buckling or fracture, particularly in polymer-based systems during manual or self-administration [54]. Conversely, increasing base diameter can improve structural integrity but may reduce insertion efficiency due to greater skin deformation and higher insertion force requirements. Similarly, needle density must be optimized to maximize drug delivery area while minimizing the “bed-of-nails” effect. The last one can hinder effective penetration, influence force distribution across the patch, and affect penetration uniformity and user-dependent variability [94]. Therefore, an optimal microneedle geometry represents a compromise between structural robustness, reliable skin insertion, and minimal tissue trauma, all of which are essential for achieving consistent drug delivery and supporting clinical translation of polymeric microneedle systems. These relationships highlight that mechanical performance must be evaluated as an integrated system property rather than an isolated material characteristic [32].

Several representative studies demonstrate how mechanical performance governs the success or failure of polymeric microneedle systems, largely independent of their chemical composition.

Kochhar et al. systematically evaluated the effect of needle height, base diameter, and tip angle on skin penetration and showed that increasing needle height beyond an optimal threshold does not necessarily improve penetration efficiency but instead increases the likelihood of buckling and fracture, particularly for polymeric microneedles with moderate elastic modulus. Their results emphasized that geometric optimization must be tailored to the mechanical properties of the selected polymer rather than maximized indiscriminately [50].

Practical limitations associated with blunt or short microneedles have been reported in investigations focusing on manual and self-administration scenarios. Gill and Prausnitz showed that microneedles with insufficient height or overly blunt tips require higher and less reproducible application forces, resulting in large inter-user variability and incomplete skin penetration [54]. These findings are particularly relevant for patch-based systems intended for home use, where controlled application forces cannot be guaranteed.

The role of tip diameter in microneedle insertion was investigated by Römgens et al., who demonstrated that tip diameter influences both the force required for skin penetration and the resulting penetration depth [92]. Beyond geometry, the magnitude of the applied force is also critical. Cheung et al. showed that insufficient insertion force may result in incomplete skin penetration and negligible insulin permeation, whereas increasing the applied force significantly enhanced insulin delivery across porcine skin [55]. Together, these findings demonstrate that reliable microneedle performance depends on the interaction between tip geometry and application force rather than on either parameter alone.

**Figure 5:**
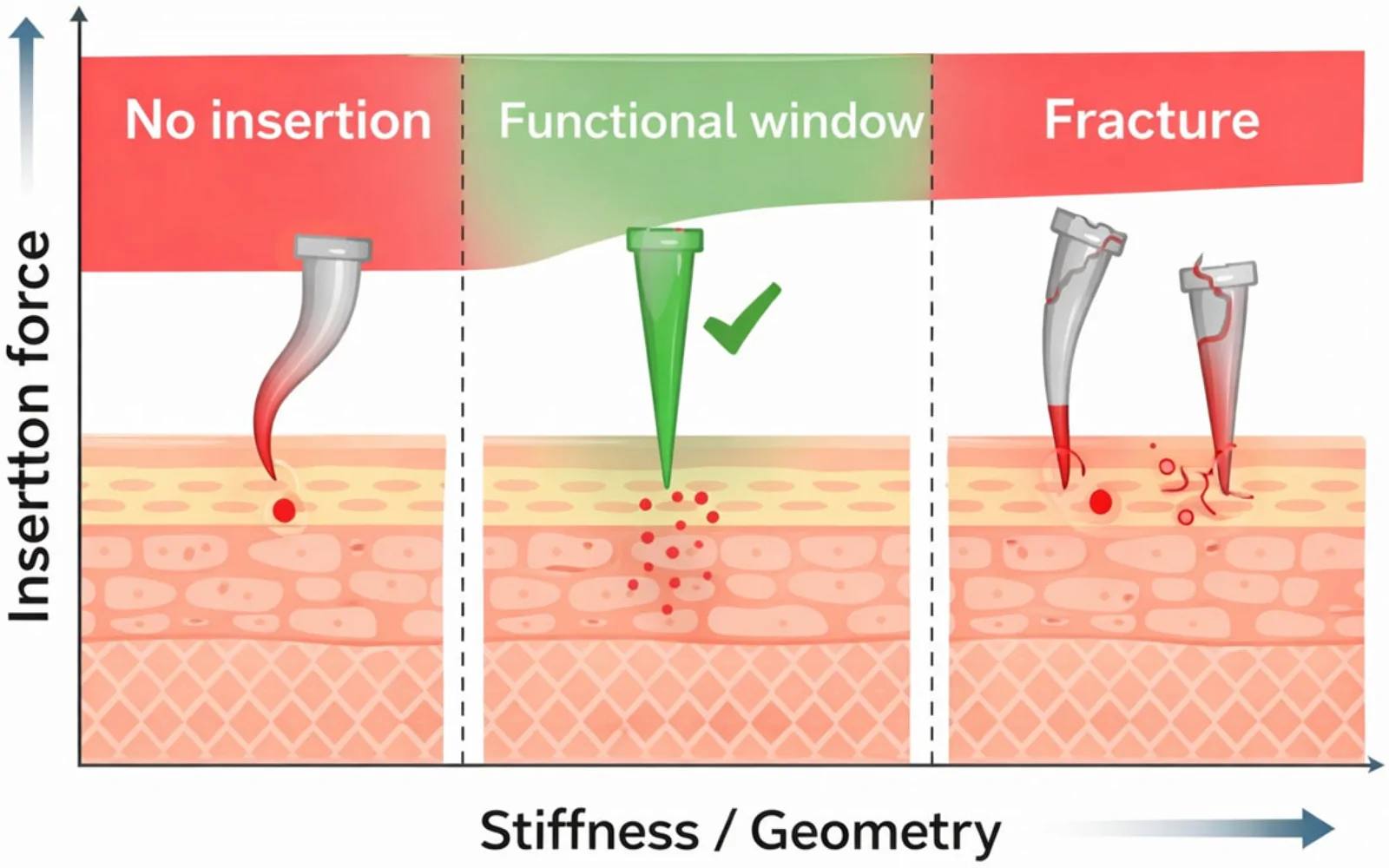
Mechanical constraints governing microneedle insertion and failure. The schematic highlights the narrow functional window required for effective microneedle performance. Insufficient stiffness results in failed insertion, whereas excessive stiffness or applied force leads to fracture. This balance defines a critical design constraint for reliable drug delivery.

Early work by Park et al. showed that biodegradable PLA and PLGA microneedles with sufficient axial fracture force could reliably penetrate murine and human skin models, provided that needle geometry and tip sharpness were appropriately optimized [43]. However, the same study revealed that mechanically robust microneedles with suboptimal tip geometry exhibited incomplete insertion despite high fracture resistance, underscoring that material strength alone is insufficient to ensure functional penetration. This apparent contradiction reflects the fact that successful skin penetration is governed not by absolute mechanical strength, but by the balance between insertion force and fracture resistance within a narrow mechanical window (Figure 5). Biodegradable polyester microneedles, including PLA- and PLGA-based systems, can achieve high axial fracture force, suboptimal tip geometry—particularly increased tip radius or a blunted apex—significantly increases the force required to breach the stratum corneum. As a result, even mechanically robust structures may fail to penetrate effectively, as the applied force is dissipated through skin deformation rather than concentrated at the needle tip. In contrast, microneedles with sharper tips and optimized aspect ratios reduce the insertion force threshold, enabling efficient penetration at forces well below the fracture limit. This interplay highlights that geometry-driven stress concentration at the needle–skin interface is a more critical determinant of functional insertion than bulk material strength alone.

Similarly, Davis et al. [32] systematically measured both parameters across microneedles with varying geometries and materials and showed that successful skin penetration occurs only within a narrow mechanical window, where the applied force exceeds the resistance of the stratum corneum but remains below the fracture threshold of the microneedle [32]. Their work revealed that increasing polymer stiffness or needle thickness beyond this window does not necessarily improve insertion efficiency and may instead promote brittle failure or uncontrolled fracture.

This behavior arises from the fundamentally different physical processes governing microneedle deformation and skin penetration. While axial fracture force reflects structural resistance to compressive failure, successful insertion depends on the generation of sufficiently high localized stress at the needle tip to overcome the barrier properties of the stratum corneum [32]. In systems with suboptimal tip geometry, the applied force is distributed over a larger contact area, reducing stress concentration and promoting skin deformation rather than penetration. Consequently, the skin behaves as a viscoelastic material that absorbs and redistributes the applied load, resulting in elastic indentation instead of barrier disruption [32].

Moreover, this mismatch is amplified by the time-dependent mechanical response of skin. Under typical application conditions, viscoelastic relaxation of the stratum corneum leads to partial dissipation of the applied force, thereby increasing the effective insertion threshold [32]. In such cases, even microneedles with high fracture resistance may fail to penetrate if the rate and mode of force application are not optimized. In contrast, microneedles with sharper tips and optimized aspect ratios promote stress localization and minimize energy dissipation, enabling efficient penetration at lower applied forces and within shorter timeframes [32].

Importantly, this highlights that insertion efficiency is not solely a function of material properties, but rather an emergent property arising from the interplay between geometry, polymer mechanics, and skin biomechanics. Representative literature-derived thresholds used to describe microneedle insertion efficiency are summarized in Supplementary Table S2 [12,68,95]; these values should be interpreted in the context of the specific material, geometry, skin model, and test conditions.

Geometric effects have been further emphasized in studies examining array configuration and interspacing. Olatunji et al. demonstrated that increasing microneedle density within an array can significantly elevate the force required for successful penetration due to load sharing across needles, even when individual needles possess sufficient mechanical strength [82]. This phenomenon has important implications for patch design, as mechanically sound microneedles may fail collectively if array-level parameters are not properly optimized [82].

This behavior reflects the transition from single-needle mechanics to collective array behavior, in which force distribution and skin deformation become dominant factors. While individual microneedles may be designed to exceed the critical insertion threshold, increasing array density distributes the applied force across multiple needles rather than concentrating it at a single tip. As a result, the effective force per needle decreases, often falling below the threshold required to overcome the mechanical resistance of the stratum corneum [82].

In addition to load sharing, closely spaced microneedles induce cooperative deformation of the skin surface, leading to increased tissue indentation and lateral stress redistribution. Rather than acting as independent penetration units, densely packed arrays interact mechanically through the underlying skin, which behaves as a compliant, viscoelastic substrate. This interaction reduces stress localization at each needle tip and increases the total force required for successful insertion. Consequently, even arrays composed of mechanically robust microneedles may fail to penetrate uniformly if interspacing is insufficient [82].

Furthermore, this phenomenon is strongly influenced by application conditions. Under manual administration, variability in applied force and insertion angle can exacerbate uneven load distribution across the array, resulting in partial insertion or heterogeneous penetration depth. In contrast, optimized interspacing promotes more independent needle action, minimizing mechanical interference and improving insertion reliability [82].

These findings underscore that microneedle performance must be evaluated at the array level rather than solely at the level of individual needles. Failure to account for these collective mechanical effects represents a key source of discrepancy between in vitro mechanical characterization and in vivo performance.

Importantly, studies employing optical coherence tomography have demonstrated that actual microneedle penetration depth depends strongly on needle geometry and application force and may remain considerably lower than the nominal needle height. Donnelly et al. showed that increasing microneedle height and application force significantly increased penetration depth in neonatal porcine skin, highlighting the importance of evaluating insertion directly in skin rather than relying exclusively on nominal dimensions [51]. These observations illustrate a recurring design limitation in which mechanical performance is assessed in isolation, without accounting for the complex viscoelastic nature of skin [52,94,96].

Importantly, mechanical performance must be evaluated in relation to the intended drug delivery application. For intradermal vaccination, partial penetration may be sufficient provided antigen deposition occurs within immune-active skin layers [54,97,98]. In contrast, microneedle systems designed for systemic delivery or long-acting implantation require deeper and more consistent penetration to ensure reliable dosing and pharmacological effect [46,99]. Failure to align mechanical design with pharmacological intent has contributed to numerous systems demonstrating promising in vitro characteristics but limited in vivo efficacy [83].

#### 5.1.2. Polymer Mechanical Properties

Polymer-related factors influencing drug delivery efficiency arise primarily from the physicochemical and mechanical characteristics of the polymeric matrix, which govern microneedle insertion, dissolution or swelling behavior, drug release kinetics, diffusion through the polymer network, and formulation reproducibility. Key determinants include polymer molecular weight, concentration, chemical structure, hydrophilicity, crosslink density, swelling capacity, dissolution rate, mechanical modulus, glass transition temperature, crystallinity, drug–polymer interactions, mesh size, degradation behavior, and residual moisture content.

Polymeric microneedles are usually fabricated from water-soluble, biodegradable and hydrogel polymers, either as single-component systems or polymer blends. Since these materials are generally mechanically weaker than silicon, metals, or ceramics, careful material selection is required. Achieving an optimal balance between strength, flexibility, and dissolution with a single material is often difficult; therefore, composite formulations are frequently used to improve both insertion performance and drug release [12]. Table 7 summarizes the principal polymer-related factors that influence the manufacturing quality, mechanical properties, drug release characteristics, and overall performance of polymeric microneedle systems.

Polymer-related parameters such as molecular weight, glass transition temperature (Tg), crystallinity, and elastic modulus directly influence microneedle stiffness and brittleness, thereby affecting insertion behavior [7,106]. Polymers with high Tg and crystallinity may provide sufficient mechanical strength but are also more prone to brittle fracture, whereas more ductile polymers may deform during insertion, reducing effective penetration depth [40]. These observations underscore that mechanical robustness alone does not guarantee functional skin insertion.

The influence of polymer-related parameters on microneedle insertion behavior has been clearly demonstrated across several commonly used synthetic systems. For instance, Park et al. reported that PLA- and PLGA-based microneedles with relatively high glass transition temperatures and semi-crystalline structures exhibited high fracture forces but were susceptible to brittle failure when fabricated with sharp tip geometries or applied under non-uniform loading conditions [43]. In these systems, mechanical strength alone did not ensure reliable skin penetration, particularly when minor geometric imperfections were present.

In contrast, dissolving microneedles fabricated from more ductile polymers such as PVP or PVA have been shown to penetrate skin effectively despite their lower elastic modulus, provided that molecular weight and needle geometry were carefully optimized. Lee et al. demonstrated that increasing the molecular weight of PVP improved microneedle stiffness sufficiently to enable insertion, while excessive ductility at lower molecular weights resulted in elastic deformation and reduced penetration depth [41]. These findings illustrate that an optimal balance between stiffness and deformability is required, rather than maximal mechanical strength.

Similar trade-offs have been demonstrated for additional polymer classes. Carboxymethylcellulose (CMC) microneedles provide an instructive example, as increasing polymer concentration improved needle formation and fracture resistance but also slowed dissolution and, at sufficiently high concentrations, promoted tip shortening and air-entrapment defects during fabrication [109,110,111]. Polycaprolactone (PCL), in contrast, has been exploited for biodegradable sustained-release microneedles because its lower degradation rate enables prolonged drug release, although this benefit must be balanced against longer polymer residence times within tissue [109,112]. Likewise, PEG-based hydrogel microneedles demonstrate that crosslink density can be tuned to balance mechanical integrity with swelling-mediated drug diffusion, illustrating that similar design trade-offs extend beyond polyester-based systems [113].

Crystallinity has also been identified as a critical determinant of microneedle failure mode, as it governs not only stiffness but also fracture behavior at the microscale [7,9]. Highly crystalline polymers typically exhibit increased elastic modulus and strength; however, their limited chain mobility results in reduced capacity for plastic deformation, leading to brittle fracture once the critical stress threshold is exceeded. In the context of microneedles, this often manifests as abrupt tip failure during insertion, particularly under conditions where stress is highly localized at the needle apex [9]. Conversely, amorphous or semi-amorphous polymers possess greater chain mobility and energy dissipation capacity, enabling more gradual deformation through viscoelastic or plastic mechanisms. However, this apparent advantage is accompanied by a different type of functional limitation. Under compressive loading, amorphous systems may undergo progressive deformation, including tip blunting, axial shortening, or lateral bending, all of which reduce the effective stress concentration required for skin penetration. As a result, insertion may still occur but in a partial and non-uniform manner, leading to heterogeneous penetration depths across the microneedle array. This phenomenon is particularly problematic in drug delivery applications, where dose accuracy depends on consistent insertion of all needles within the array.

Importantly, the transition between brittle fracture and plastic deformation is not solely dictated by intrinsic polymer structure but is also influenced by environmental and formulation-dependent factors, including temperature, hydration, and plasticizer content [7]. For example, exposure to moisture can significantly reduce the glass transition temperature of amorphous polymers, shifting their behavior toward a more deformable state during application. Consequently, microneedles fabricated from nominally rigid materials may exhibit time- and condition-dependent mechanical responses in vivo [114].

These findings highlight that crystallinity-driven mechanical behavior must be carefully balanced to avoid both catastrophic fracture and excessive deformation. An optimal microneedle design requires a controlled combination of stiffness and toughness that allows sufficient resistance to compressive loading while maintaining the ability to dissipate stress without failure. Failure to achieve this balance results either in brittle tip fracture or in progressive deformation, both of which ultimately compromise penetration efficiency and drug delivery performance.

Taken together, these examples demonstrate that polymer molecular characteristics influence not only microneedle strength but also the dominant failure mechanism during skin insertion. Consequently, mechanical robustness assessed in isolation is insufficient to predict functional performance. Instead, polymer selection must be guided by how molecular weight, glass transition temperature, and crystallinity collectively shape the balance between stiffness and brittleness under realistic insertion conditions.

#### 5.1.3. Skin- and Application-Related Factors

In addition to material and geometric factors, the therapeutic performance of polymeric microneedles is strongly influenced by application-related variables, including the mode of administration, applied force, residence time, anatomical site, skin condition, patch fixation, and user-dependent handling, all of which can affect insertion efficiency, drug release, absorption, and dose reproducibility. The following table (Table 8) summarizes the principal application-related factors, their main effects on insertion performance, drug release, absorption as well as the preferred conditions required for efficacy and reproducibility of polymeric microneedle delivery.

**Figure 6:**
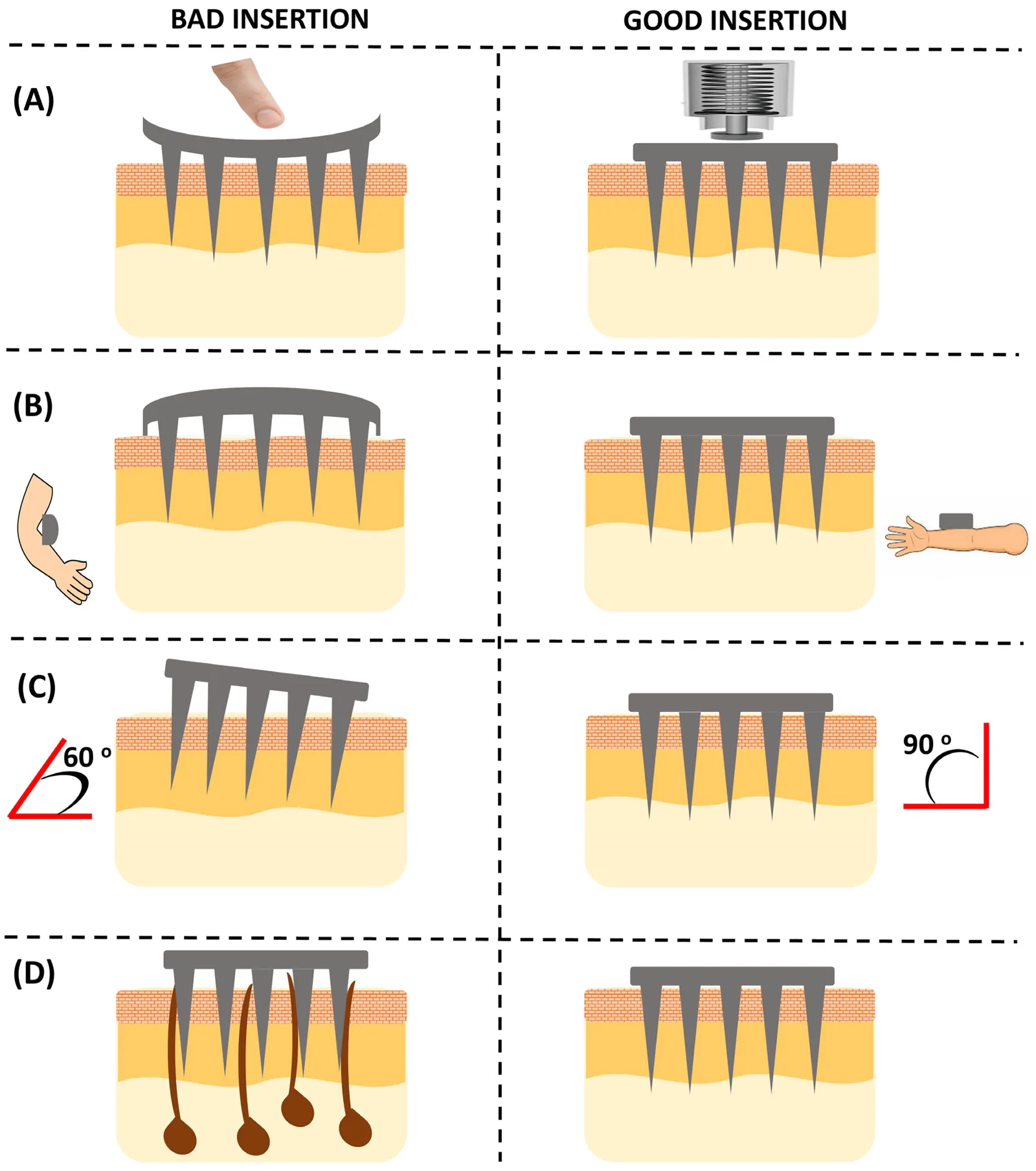
Representative examples of application-related factors influencing microneedle insertion performance. Schematic comparison of suboptimal and optimized application conditions, including: application mode—manual finger application vs. applicator-assisted application (**A**); patch fixation and adhesion during wear—excessive skin deformation vs. stable fixation (**B**); application angle—oblique vs. perpendicular insertion (**C**); skin condition before application—hairy vs. non-hairy skin (**D**). The illustrated scenarios are conceptual representations based on reported application-related mechanisms in the literature.

Overall, the efficiency and reproducibility of polymeric microneedle-mediated drug delivery are strongly influenced by application-related factors in addition to the intrinsic properties of the microneedle system. Standardized application using an appropriate applicator, controlled insertion force and angle, and a formulation-specific residence time can minimize user-dependent variability and promote consistent skin penetration and drug deposition. The condition and preparation of the skin, including its integrity, hydration, cleanliness, and surface characteristics, should also be carefully controlled, while adequate patch adhesion and minimal movement during wear are essential for maintaining effective skin–microneedle contact. Anatomical site selection should be tailored to the microneedle dimensions, target tissue, drug, and intended local or systemic delivery profile. Environmental conditions and post-application handling may further affect polymer behavior, adhesion, and delivery duration. Thus, rather than defining a single universally optimal application protocol, effective administration of polymeric microneedles requires a standardized, device- and formulation-specific approach that considers the interaction between application technique, skin characteristics, and intended therapeutic outcome. The practical influence of key application-related factors on microneedle insertion performance is summarized schematically in Figure 6.

Although standardized axial compression tests provide an essential measure of structural robustness, manual microneedle application involves dynamic loading conditions that differ substantially from quasi-static laboratory testing. Contemporary computational studies increasingly address this gap by combining finite element analysis with transient contact simulations that incorporate loading rate, impact velocity, skin deformation, and viscoelastic tissue behavior during insertion. Rather than relying solely on peak applied force, these models evaluate force–time profiles, stress distribution at the needle tip, and progressive skin deformation to predict successful penetration under realistic application conditions. Experimental studies have further complemented these simulations by measuring user-generated force profiles during manual insertion, demonstrating considerable inter-user variability in loading rate and peak force despite similar average insertion outcomes. Together, these approaches indicate that insertion success depends not only on fracture resistance but also on the dynamic interaction between needle geometry, polymer mechanical properties, skin biomechanics, and application kinetics. Consequently, computational modeling of dynamic insertion provides an important bridge between standardized mechanical characterization and the more variable loading conditions encountered during practical use [64,129].

Taken together, these findings emphasize that mechanical performance should be regarded as a functional design constraint rather than a material property alone. Rational development of polymeric microneedles requires integrated optimization of polymer characteristics, microneedle geometry, and application conditions to achieve reproducible skin penetration. Without such alignment, even advanced polymer formulations may fail to deliver therapeutically meaningful outcomes, undermining the potential of microneedles as a viable alternative route for drug delivery.

Once successful skin penetration has been achieved, the therapeutic effectiveness of microneedle systems depends on the subsequent release of the drug payload. The temporal profile of drug release must therefore be carefully aligned with the intended pharmacological objective, as discussed in the following section.

### 5.2. Pharmacokinetic–Dose Performance

Pharmacokinetic–dose performance represents the ability of a polymeric microneedle system to deliver a therapeutically effective drug exposure by achieving the appropriate dose, release profile, and absorption at the target site. It encompasses the interplay between the physicochemical properties of the encapsulated drug, drug loading capacity, release behavior, skin transport processes, and systemic pharmacokinetic characteristics, all of which collectively determine the amount, rate, and duration of drug delivery.

#### 5.2.1. Physicochemical Properties of the Incorporated Drug

The physicochemical properties of the encapsulated drug are critical determinants of drug delivery efficiency from polymeric microneedle systems, as they influence drug incorporation within the polymeric matrix, its stability during manufacturing and storage, release from the microneedles, and subsequent partitioning and permeation through the skin. Parameters such as molecular size, aqueous solubility, lipophilicity, ionization state, solid-state properties, and drug–polymer interactions can substantially affect these processes and may ultimately determine the fraction of the loaded dose that reaches the intended tissue. Moreover, the required therapeutic dose and drug loading capacity can impose additional formulation constraints, particularly because the amount of drug that can be incorporated into microneedle arrays is often limited. Consequently, successful design of polymeric microneedles requires careful matching of the physicochemical characteristics of the encapsulated drug with the properties of the polymer matrix and the intended route and site of delivery. The major physicochemical properties of the encapsulated drug that may influence drug loading, release, skin permeation, and overall delivery efficiency of polymeric microneedles are summarized in Table 9.

#### 5.2.2. Release Programming: Aligning Polymer Behavior with Pharmacological Intent

Drug release from polymeric microneedles should be regarded as a primary functional design parameter rather than a passive consequence of polymer selection. The pharmacological objective—whether rapid local anaesthesia, sustained systemic exposure, or controlled immunization—must dictate the release architecture, not the other way around. Failure to align release kinetics with therapeutic intent represents one of the most frequently overlooked translational bottlenecks in the field [43,99].

From a mechanistic perspective, drug release from polymeric microneedles is governed by three primary processes: polymer dissolution, polymer degradation, and diffusion through hydrated matrices. In dissolving systems, release is dominated by rapid polymer dissolution, whereas in biodegradable systems such as PLGA, drug liberation is controlled by a combination of diffusion and hydrolytic degradation. In hydrogel-forming systems, transport occurs primarily via diffusion through a swollen polymer network, often coupled with reservoir-based delivery. Recognizing these distinct mechanisms is essential for rational release design, as each imposes specific constraints on drug loading, stability, and achievable release kinetics.

The alignment between therapeutic objectives, release profiles, and polymer selection is summarized in Table 10, illustrating how different microneedle designs should be tailored to specific pharmacological requirements.

As shown in Table 10, no single release strategy is universally optimal; rather, the suitability of a given system depends on its ability to match the temporal requirements of the intended therapeutic application.

The principal release strategies employed in polymeric microneedle systems can be broadly categorized as immediate, biphasic, and sustained, each associated with distinct polymer choices, fabrication strategies, and pharmacological applications. Dissolving microneedles fabricated from rapidly water-soluble polymers such as poly(vinylpyrrolidone) (PVP) and poly(vinyl alcohol) (PVA) typically produce immediate or near-instant release profiles. Sullivan et al. demonstrated that PVP-based microneedles can deliver protein cargo within minutes of skin insertion, exploiting the high aqueous solubility and rapid dissolution of the polymer matrix [40]. This approach is well suited to vaccination, where rapid antigen deposition in immunologically active skin layers is desirable, but is inappropriate for indications requiring sustained systemic exposure [41].

In contrast, poly(lactic-co-glycolic acid) (PLGA)-based systems offer tunable sustained release through hydrolytic degradation, with release durations ranging from hours to months depending on monomer ratio, molecular weight, and encapsulation strategy. Park et al. demonstrated that PLGA microneedles can achieve controlled release either through direct drug incorporation into the polymer matrix or via encapsulation within PLGA microparticles, the latter introducing a two-stage release mechanism that prolongs delivery [43]. PLGA with an approximately 50:50 lactide-to-glycolide ratio generally undergoes faster degradation than copolymers with a higher lactide content, allowing polymer composition to be selected according to the intended release duration [9,133].

Biphasic release profiles, combining an initial rapid phase with prolonged delivery, have been achieved through multilayer microneedle architectures. For example, PLGA–PVP bilayer systems enable rapid dissolution of the outer PVP layer to deliver a bolus dose, followed by sustained release from the PLGA core [136]. While such systems are conceptually attractive for indications requiring both rapid onset and prolonged action, their increased fabrication complexity introduces reproducibility challenges and may limit scalability [99].

Beyond system architecture, the physicochemical properties of the drug payload impose additional constraints on release strategy selection. Small hydrophilic molecules diffuse readily through dissolving matrices, making burst-release systems suitable for many low-molecular-weight drugs. In contrast, biologics such as proteins, peptides, and nucleic acids exhibit restricted diffusion within dense polymer networks and are susceptible to degradation during fabrication, particularly in acidic microenvironments generated by PLGA hydrolysis. These factors necessitate careful polymer selection to preserve drug stability and functionality [82,93]. A critical but often underappreciated limitation in release characterization is the discrepancy between in vitro and in vivo conditions. Standard in vitro assays, typically conducted under sink conditions with constant agitation, do not replicate the complex environment of the skin, where limited interstitial fluid volume, heterogeneous tissue structure, and variable perfusion significantly influence drug transport. Consequently, release profiles that appear well controlled in vitro may not translate into predictable pharmacokinetic behavior in vivo. This discrepancy underscores the need for physiologically relevant testing models and robust in vitro–in vivo correlation strategies.

Appropriate evaluation of release behavior requires selecting analytical methods according to the intended question. In vitro release testing (IVRT) is primarily designed to characterize formulation-specific drug release under standardized conditions and is not intended to reproduce the physiological skin environment. By contrast, in vitro permeation testing (IVPT) evaluates drug transport across a biological or synthetic barrier, thereby providing greater physiological relevance for predicting transdermal performance. Nevertheless, translational assessment should not rely on IVPT alone. A comprehensive evaluation of polymeric microneedles typically combines IVRT to characterize release kinetics, IVPT to assess permeation, ex vivo studies using human or porcine skin to evaluate insertion and transport under realistic tissue conditions, and in vivo investigations to confirm pharmacokinetic performance and therapeutic efficacy [35,38,63]. From a function-driven perspective, the evaluation method should be aligned with the development stage: IVRT primarily supports formulation optimization, IVPT and ex vivo skin studies evaluate functional delivery performance, whereas in vivo studies ultimately establish clinical relevance.

Complementary in vivo sampling approaches, including dermal microdialysis and dermal open-flow microperfusion (dOFM), can provide direct information on local dermal drug exposure. Microdialysis enables continuous sampling of unbound analytes through a semipermeable probe, whereas the membrane-free design of dOFM permits sampling across a broader range of molecular sizes and physicochemical properties. Although these techniques are not routinely required during early microneedle development, they can provide valuable cutaneous pharmacokinetic information during later translational evaluation [137].

A recurring design misalignment in the literature concerns systems exhibiting well-defined in vitro release profiles that are poorly matched to clinical pharmacokinetic requirements. As reflected in Table 10, failure to align release kinetics with pharmacological needs remains a major barrier to translation. Koyani noted that many microneedle studies report favorable in vitro release without pharmacokinetic validation or therapeutic endpoint data, limiting their clinical relevance. Similarly, Prausnitz and Langer highlighted that only a limited number of drugs have been successfully translated into transdermal delivery systems, reflecting the stringent constraints imposed by the skin barrier [138]. Recent analyses further indicate that clinically successful microneedle systems require simultaneous optimization of release kinetics, skin permeation, manufacturability, and user-dependent performance, reinforcing that release programming should be considered within a broader translational framework rather than as an isolated formulation attribute [38,63,65].

These observations demonstrate that successful release design in polymeric microneedles is not a material-driven outcome but a function-driven process requiring explicit alignment between drug properties, polymer behavior, and therapeutic objectives.

In addition to release behavior, an equally important consideration is the fate of the polymeric matrix following microneedle insertion. Whether the material dissolves, degrades, or remains within the skin has significant implications for safety, efficacy, and regulatory acceptance.

#### 5.2.3. Polymer Fate in the Skin: Dissolution, Implantation, and Accumulation

The fate of polymers following microneedle insertion represents a critical yet systematically underreported aspect of microneedle design. While mechanical performance and release programming have received extensive attention, the post-delivery behavior of the polymeric matrix is rarely examined with the rigor required for safety evaluation, particularly in the context of repeated or chronic administration [52,99].

**Figure 7:**
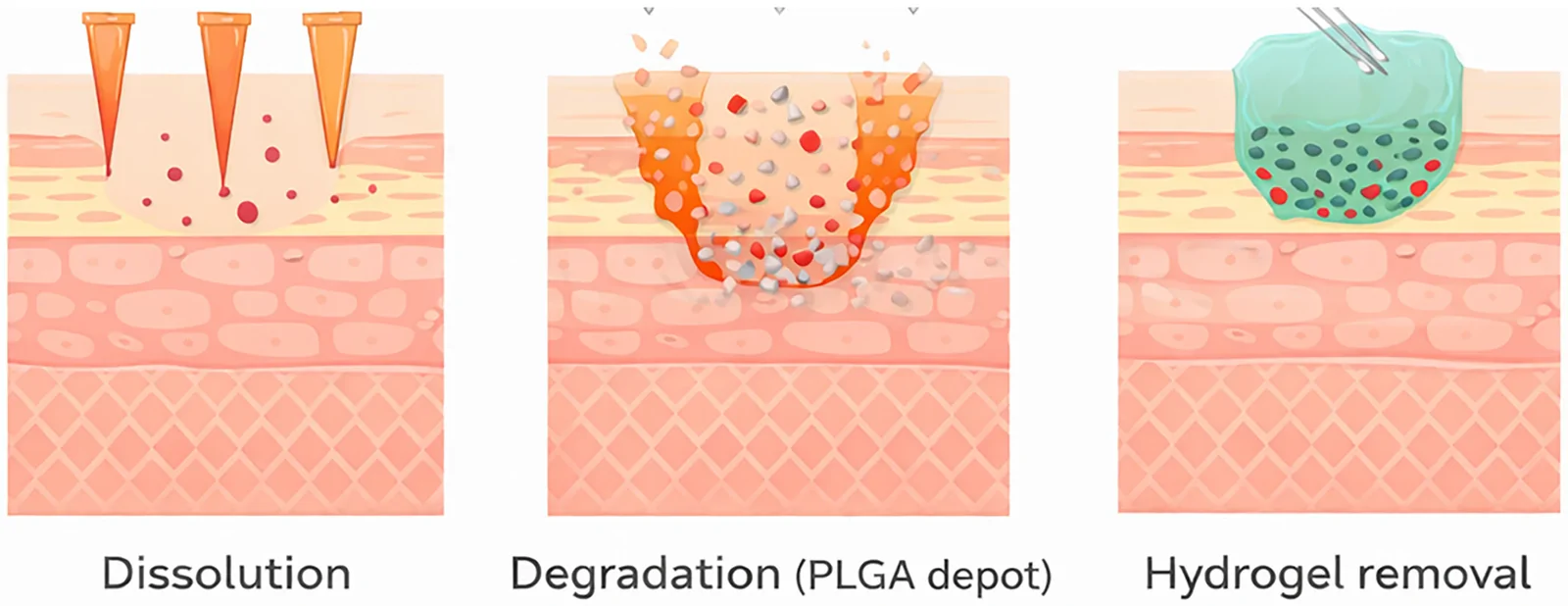
Polymer fate in the skin. Illustration of polymer dissolution, intradermal degradation, and hydrogel removal following microneedle insertion.

From a functional and safety perspective, polymer fate in microneedle systems can be categorized into three principal scenarios: (i) complete dissolution and clearance (e.g., PVP, PVA systems), (ii) temporary residence followed by degradation (e.g., PLGA-based systems), and (iii) structural retention with subsequent removal (e.g., hydrogel-forming systems). The three principal scenarios governing polymer fate in the skin are schematically illustrated in Figure 7. Each scenario is associated with a distinct safety profile and regulatory burden, and should therefore be considered a primary design parameter rather than a secondary consequence of material selection.

Dissolving microneedles fabricated from rapidly water-soluble polymers such as PVP and PVA undergo complete dissolution within seconds to minutes upon contact with interstitial fluid, leaving no residual material in the tissue. Koyani reported dissolution times ranging from 10 s to 15 min depending on composition, with PVP-based systems generally dissolving more rapidly due to their higher hygroscopicity [99]. For single-use applications such as vaccination, this rapid clearance is both pharmacologically and toxicologically acceptable, given the low polymer dose and limited tissue exposure.

The situation changes substantially for systems designed for sustained drug delivery, in which the polymer must persist within the skin to fulfil its release function. PLGA-based microneedles have been developed for intradermal implantation, with drug-loaded tips designed to remain in the tissue for weeks to months [77,139]. While this property enables sustained release, it also results in the local accumulation of polymer and its degradation products—lactic and glycolic acids—throughout the release period. Although PLGA is an FDA-approved biomaterial with an established safety record in implants, systematic dermal safety data under conditions of repeated microneedle administration remain limited, representing an important regulatory gap [52,93].

In addition to polymer accumulation, the local microenvironment generated during degradation plays a critical role in determining tissue response. The acidification associated with PLGA hydrolysis may alter enzymatic activity, protein stability, and cellular behavior, particularly under repeated dosing conditions where local buffering capacity may be exceeded. While these effects are well characterized in bulk implant systems, they remain insufficiently explored in the context of distributed intradermal microneedle depots.

Future studies would benefit from adopting standardized multimodal evaluation strategies capable of quantifying both localized tissue acidification and its biological consequences following repeated administration of biodegradable polymeric microneedles. Direct assessment of tissue pH using microelectrode measurements or pH-sensitive fluorescent probes can provide spatially resolved information on local acidification, while chromatographic techniques such as high-performance liquid chromatography (HPLC) or liquid chromatography–mass spectrometry (LC–MS) enable quantitative determination of lactic and glycolic acid accumulation within treated tissues. These analytical measurements should be complemented by histological evaluation using hematoxylin and eosin (H&E) staining to assess tissue architecture and inflammatory cell infiltration, together with immunohistochemical detection of macrophage markers (CD68), neutrophil markers (Ly6G), and pro-inflammatory cytokines such as TNF-α, IL-1β, and IL-6. Additional multiplex cytokine assays or ELISA-based quantification of inflammatory mediators can further distinguish transient tissue responses from persistent inflammatory reactions. Collectively, these complementary approaches would provide a more comprehensive understanding of how polymer degradation, local pH changes, and host immune responses interact during repeated microneedle administration and could facilitate the development of standardized safety evaluation protocols for biodegradable polymeric microneedle systems [52,77,93,139].

An additional dimension of polymer fate relates to the microchannels created during microneedle insertion. Microneedle-created microchannels generally reseal within several hours, although the closure time varies considerably depending on microneedle geometry, skin condition, and whether the application site is occluded [10]. This resealing kinetics defines the window of increased susceptibility to infection and has direct implications for repeated-use safety. For systems intended for chronic application, the cumulative effect of repeated microchannel formation at the same anatomical site warrants systematic evaluation.

Hydrogel-forming microneedles represent a distinct delivery strategy in which the crosslinked polymer matrix absorbs interstitial fluid and swells without dissolving in the skin, thereby enabling drug diffusion from an attached reservoir. The swollen microneedle array is intended to remain mechanically intact and to be removed after use [11,30,131]. Consequently, its development requires evaluation of local biocompatibility, irritation and sensitization potential, mechanical integrity after swelling, and the possibility of polymer fragment retention, particularly under repeated-use conditions. Such evaluations should follow the applicable parts of the ISO 10993 series, including ISO 10993-23 for irritation [140] and ISO 10993-10 for skin sensitization [141]. A further, often overlooked factor is the spatial distribution of polymer material within the skin. Unlike conventional implants, microneedles generate a distributed pattern of microdeposits across the dermis. This dispersion may influence degradation kinetics, clearance pathways, and local immune response, as heterogeneous polymer distribution can lead to localized concentration gradients and variable tissue exposure.

The risk of polymer accumulation becomes particularly relevant in repeated-use scenarios. Koyani emphasized that the long-term consequences of cumulative polymer deposition following repeated microneedle application remain poorly characterized, and that adverse outcomes such as granuloma formation or chronic inflammation cannot be excluded based on current evidence [99,142]. Similarly, Waghule et al. identified retained microneedle fragments as a potential safety concern in cases of mechanical failure during insertion, particularly for slowly degrading synthetic polymers. Bonfante et al. further showed that material shrinkage during fabrication may introduce internal stress, increasing the likelihood of tip fracture—a phenomenon that, although reported for polysaccharides, is also relevant for synthetic systems undergoing volumetric changes during processing [106]. Interindividual variability in skin properties represents an additional layer of complexity in predicting polymer fate. Factors such as age, hydration, skin thickness, and vascularization may influence polymer degradation, clearance, and local tissue response, potentially contributing to variability in both drug delivery and safety outcomes. These variables are rarely incorporated into preclinical studies but may become critical determinants of clinical performance.

From a regulatory perspective, the fate of the polymeric component must be evaluated alongside the pharmacological payload within the overall safety dossier. Therefore, approval requires robust evidence that microneedle-induced skin disruption does not result in persistent tissue damage or an unacceptable infection risk [6,7]. For synthetic polymer systems, this requirement extends to demonstrating the biocompatibility of both the intact material and its degradation products under realistic conditions of use—a requirement that remains insufficiently addressed in the current literature and represents a significant barrier to clinical translation.

The interplay between material behavior, mechanical performance, and release characteristics ultimately determines whether a microneedle system can achieve clinically relevant outcomes. However, despite promising experimental data, many systems fail to progress toward clinical application, as discussed in the following section. These considerations highlight that polymer fate should be regarded as a critical determinant of long-term safety rather than a secondary outcome of material choice.

Although numerous polymeric microneedle systems have demonstrated promising preclinical performance, successful clinical translation depends on overcoming challenges beyond drug delivery efficiency alone. Representative examples of commercial and clinically investigated microneedle platforms (Table S3) illustrate how manufacturing robustness, user interaction, device usability, and regulatory considerations influence progression toward clinical implementation.

While conventional microneedle systems rely on predefined release profiles, increasing attention has been directed toward systems capable of dynamic, stimuli-responsive drug delivery, in which release is triggered by physiological or external cues.

## 6. Responsive and Smart Microneedle Systems: Added Value or Added Complexity?

The emergence of stimuli-responsive microneedle systems represents one of the most actively pursued directions in the field, driven by the appeal of on-demand drug release triggered by physiological or external stimuli. However, despite their conceptual appeal, a critical appraisal indicates that responsiveness does not invariably translate into clinical added value and often introduces fabrication complexity, reproducibility challenges, and safety concerns that may hinder rather than facilitate translation [50,99].

**Figure 8:**
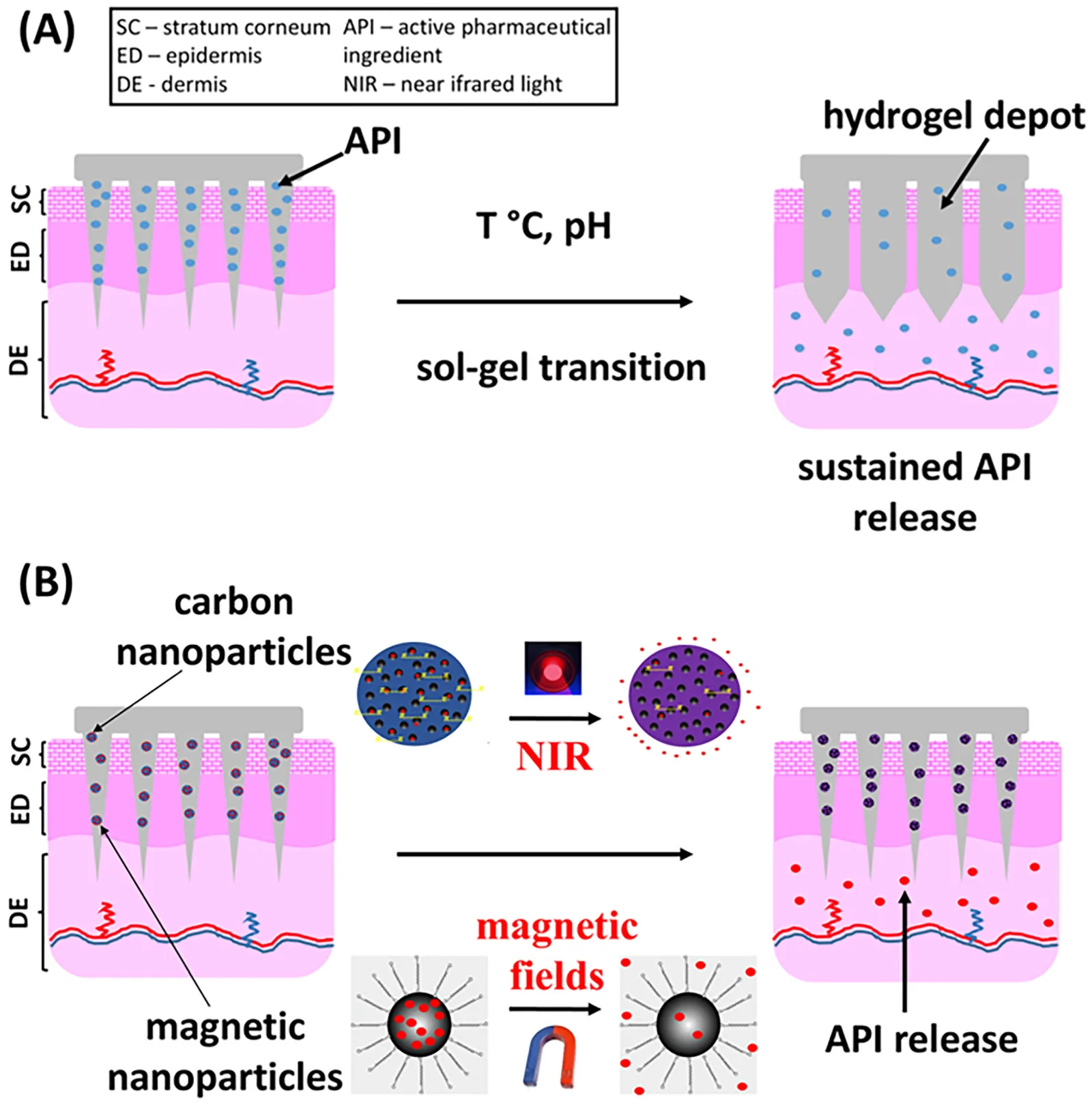
Stimuli-responsive polymeric microneedles based on activation mechanisms. Schematic representation of internally triggered systems, which respond to physiological cues such as pH, temperature, and enzymes (**A**), and externally triggered systems, which are activated by external stimuli such as near-infrared (NIR) irradiation and magnetic fields (**B**).

From a functional standpoint, stimuli-responsive systems can be broadly categorized into internally triggered systems (e.g., pH-, glucose-, or enzyme-responsive) and externally triggered systems (e.g., near-infrared light irradiation, NIR or magnetic fields), as visualized in Figure 8. This distinction has direct implications for clinical applicability: internally triggered systems are inherently more compatible with autonomous drug delivery, whereas externally triggered systems require user intervention and device integration, potentially limiting their practicality in routine clinical use.

Among thermoresponsive systems, poly(N-isopropylacrylamide) (PNIPAm)-based microneedles have attracted particular attention due to their lower critical solution temperature near physiological conditions. Li et al. developed rapidly separable thermosensitive microneedles based on gelatin-g-PNIPAm, which undergo a temperature-induced gel-to-sol transition at physiological temperature, enabling rapid deposition of the insulin-loaded component into the skin followed by controlled drug release [134]. In diabetic animal models, this system produced slower and more prolonged glucose reduction compared to non-modified gelatin microneedles. However, its fabrication required multi-step synthesis, crosslinking optimization, and a complex rapidly separating architecture, raising questions about whether such complexity provides clinically meaningful advantages over simpler sustained-release systems based on PLGA or PVP [46,99]. Externally triggered systems, including NIR- and magnetically responsive microneedles, have also been explored for on-demand drug delivery. For example, PCL-based microneedles incorporating photothermal nanostructures (like carbon nanoparticles) enable drug release upon laser irradiation, while Fe_3_O_4_-loaded systems allow modulation via alternating magnetic fields [99]. Although these approaches demonstrate proof-of-concept responsiveness, their clinical applicability is constrained by the requirement for specialized external devices, which limits patient self-administration and compatibility with standard clinical settings. In addition, the long-term safety of retained inorganic nanomaterials within the skin remains insufficiently characterized.

Looking beyond current hardware limitations, artificial intelligence (AI) may contribute to the future implementation of stimuli-responsive microneedle systems by improving how external energy-delivery devices are controlled rather than replacing them. Emerging AI-assisted approaches—including machine-learning algorithms for treatment optimization, closed-loop control using real-time sensor feedback, and digital-twin-assisted treatment planning—could enable individualized adjustment of irradiation time, magnetic field intensity, or stimulation frequency according to patient-specific tissue characteristics and therapeutic responses. In parallel, AI-driven image analysis may support automated assessment of microneedle insertion quality and treatment consistency, while predictive models may help optimize stimulation protocols during device development. Nevertheless, these approaches remain largely at the preclinical or early translational stage, and the requirement for external NIR or magnetic stimulation hardware continues to represent a practical limitation for routine clinical implementation. Consequently, AI should currently be viewed as a complementary technology capable of enhancing the precision, personalization, and usability of stimuli-responsive microneedle systems rather than eliminating the need for specialized activation devices [143,144].

Glucose-responsive microneedle systems for closed-loop insulin delivery represent one of the most clinically relevant subclasses of smart systems. Luo et al. developed a microneedle-array patch incorporating pH-sensitive insulin-loaded nanoparticles together with glucose oxidase- and catalase-loaded nanoparticles. Under hyperglycaemic conditions, glucose oxidation induces a local decrease in pH, triggering nanoparticle dissociation and insulin release. The system maintained blood glucose levels within the normal range for a prolonged period in a type 1 diabetic mouse model [145]. While the pharmacological rationale is compelling, translation remains limited by challenges including enzyme stability, sensor calibration drift, and the risk of hypoglycaemia due to unintended activation. To date, glucose-responsive microneedle systems remain at the preclinical stage, and their translation is limited by challenges related to trigger reliability, dose accuracy, enzyme stability, and hypoglycaemia risk [135]. A comprehensive analysis of both glucose oxidase-containing and phenylboronic acid-containing microneedle systems concluded that, despite their mechanistic elegance, significant challenges—including enzyme stability, dose accuracy, and hypoglycaemia risk—must be resolved before clinical translation can be considered feasible [135].

A critical determinant of the clinical viability of responsive systems is the reliability and specificity of the triggering mechanism under physiological conditions. Many systems demonstrate clear responsiveness in vitro; however, physiological stimuli in vivo—such as local pH or temperature variations—are often modest and may be insufficient to consistently activate release. This raises the risk of under-dosing due to incomplete activation or unintended release caused by non-specific environmental fluctuations.

A key distinction must therefore be made between responsive systems that address a genuine clinical need—such as glucose-triggered insulin delivery or infection-responsive antibiotic release—and those that introduce responsiveness primarily as a conceptual or technological novelty. While complexity is not inherently undesirable, it must be justified by a clear pharmacological advantage. For example, biphasic release architectures are appropriate when both rapid onset and sustained exposure are required, as in hormonal contraception or post-operative pain management [43]. However, in the absence of such justification, additional system complexity may reduce robustness, reproducibility, and translational feasibility.

Within this function-driven framework, over-engineered microneedle systems that are insufficiently aligned with biological and clinical requirements may introduce additional manufacturing, safety, and regulatory uncertainties without providing a corresponding therapeutic benefit [12]. Stimuli-responsiveness should therefore be implemented only when it directly addresses a clearly defined therapeutic requirement. Otherwise, it risks transforming potentially viable delivery systems into unnecessarily complex and clinically impractical constructs.

These observations demonstrate that translational failure in microneedle systems arises not from isolated technical limitations, but from systemic misalignment between design, application, and clinical reality. Overcoming this gap requires a shift from technology-driven innovation toward rigorously validated, function-oriented system design.

The recurring design–performance mismatches identified above highlight the need for a more systematic approach to microneedle development. These observations form the basis for the function-driven design principles outlined in the following section.

## 7. Function-Driven Development Framework: Comparing Design Approaches and Optimizing the Microneedle Design Space

The preceding sections demonstrate that successful polymeric microneedle development depends on the coordinated optimization of multiple interconnected variables rather than on the selection of a single optimal polymer or fabrication method. Although numerous studies report improvements in individual formulation parameters, such as polymer composition, needle geometry, or fabrication technology, these variables are often optimized independently. This sequential optimization frequently leads to design–performance mismatches that become evident only during later stages of development, when drug delivery efficiency, manufacturability, or clinical usability are evaluated.

From a practical development perspective, three distinct design philosophies can be identified within the microneedle literature: material-driven, technology-driven, and function-driven development. While the first two approaches have generated substantial innovation, they differ fundamentally in how design decisions are prioritized throughout product development. Comparing these approaches helps explain why many technically successful microneedle systems still encounter translational barriers and illustrates how a function-driven strategy can better integrate therapeutic objectives with engineering decisions [6,9,32,40,46,50,52,54,82,93,99].

### 7.1. Systematic Comparison of Microneedle Development Approaches

Unlike conventional reviews that compare microneedle types according to structure or material composition, a comparison of development workflows highlights how design decisions are organized throughout product development. The principal characteristics of the three dominant development approaches are summarized in Table 11.

Historically, much of the microneedle literature has followed a material-driven workflow, in which a new polymer is first selected, fabricated into microneedles, mechanically characterized, and only subsequently evaluated for drug delivery performance. This strategy has successfully expanded the range of usable polymers—including PVP, PVA, hyaluronic acid, CMC, PLGA, and PCL—but often treats therapeutic performance as a downstream validation step rather than an initial design criterion.

A second development philosophy has emerged alongside advances in additive manufacturing. Technology-driven development prioritizes fabrication capabilities such as stereolithography (SLA), digital light processing (DLP), continuous liquid interface production (CLIP), and advanced micromolding. These approaches provide exceptional control over needle geometry and manufacturing precision but may still encounter translational limitations when drug release behavior, polymer degradation, or user-dependent application variability are addressed only after manufacturing optimization.

In contrast, a function-driven workflow reverses the conventional sequence of decisions. Rather than beginning with the available polymer or fabrication technology, development starts by defining the therapeutic objective and the desired pharmacological outcome. Polymer selection, needle geometry, fabrication strategy, release programming, and application conditions are subsequently optimized as interconnected variables within a predefined performance framework. This integrated strategy better reflects the recurring design–performance relationships identified throughout this review.

The flowcharts for different approach types are presented in Figure S1.

### 7.2. Decision-Making Algorithm for Function-Driven Microneedle Development

The comparison of development philosophies demonstrates that the principal advantage of a function-driven strategy lies in its ability to establish the design space before individual formulation variables are optimized. Instead of treating polymer selection, geometry, and fabrication as independent decisions, these variables become coordinated responses to predefined therapeutic requirements.

**Figure 9:**
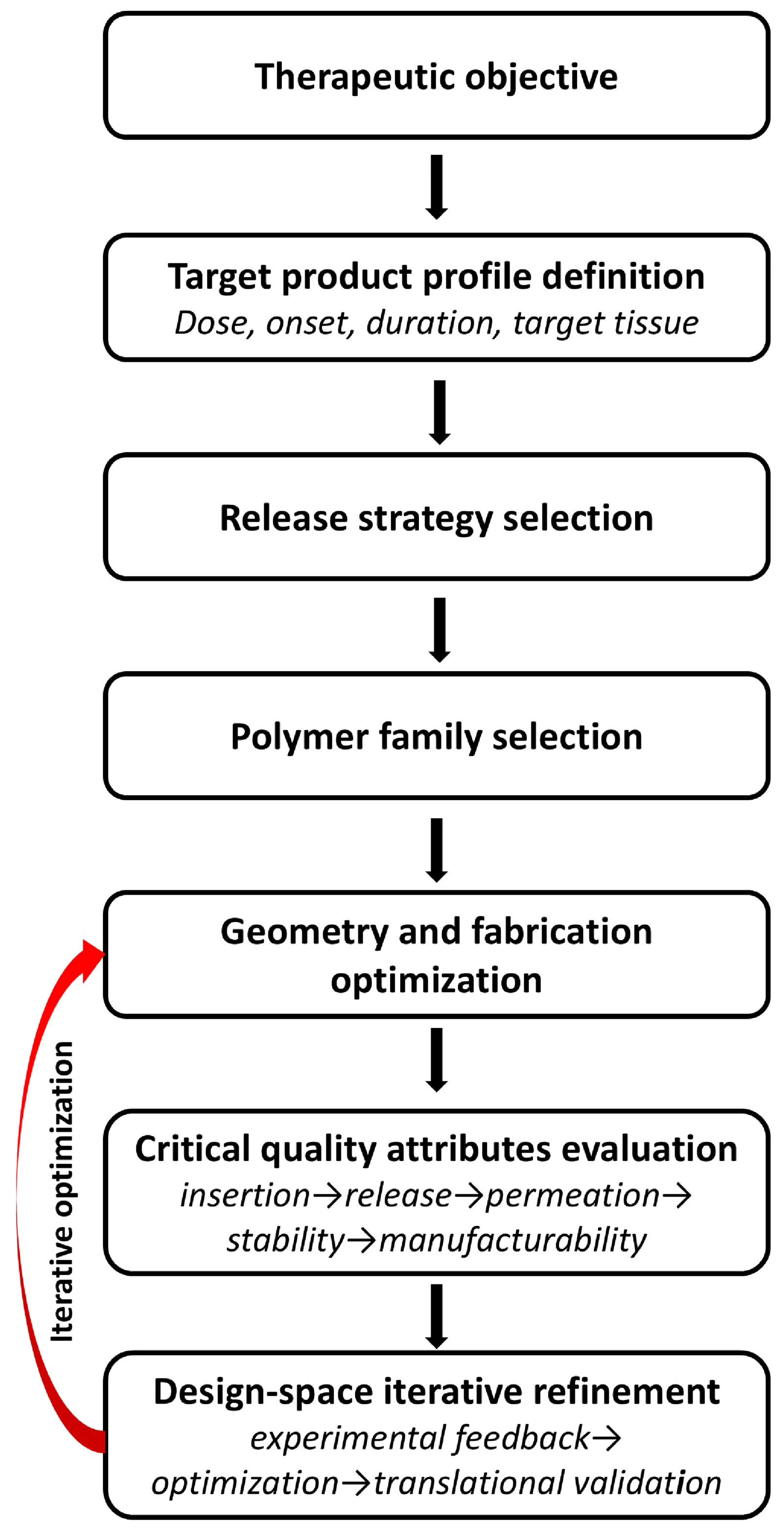
Function-driven decision algorithm for polymeric microneedle development. Therapeutic objectives define the target product profile and subsequently guide release strategy, polymer selection, geometric optimization, fabrication strategy, evaluation of critical quality attributes, and iterative refinement of the design space toward clinically viable systems.

The proposed decision algorithm, presented in Figure 9, therefore begins by defining the Target Product Profile (TPP), including the intended indication, required dose, onset of action, duration of therapy, anatomical target, and expected mode of administration. These therapeutic requirements subsequently determine the preferred release strategy, which narrows the selection of suitable polymer families, geometric parameters, and fabrication technologies.

Importantly, optimization is not completed after the first successful prototype. Experimental feedback continuously refines the acceptable operating window by evaluating critical quality attributes such as insertion performance, fracture resistance, drug release, permeation, stability, and manufacturability. This iterative process resembles contemporary Quality-by-Design (QbD) principles while remaining specifically adapted to the unique requirements of polymeric microneedle systems.

### 7.3. Establishing and Optimizing the Microneedle Design Space

The concept of design space extends beyond identifying individually favorable formulation variables. In polymeric microneedles, acceptable performance emerges only when multiple design variables operate simultaneously within a coordinated operating window. Consequently, establishing the design space, as presented in Table 12, requires identifying the variables that most strongly influence critical quality attributes and selecting optimization strategies appropriate for each development stage.

Establishing the design space requires identifying not only the individual variables that influence performance but also the interactions between them. In polymeric microneedles, needle height, base diameter, tip radius, tip angle, aspect ratio, polymer concentration, crosslink density, fabrication conditions, and application parameters frequently interact in a nonlinear manner, making sequential one-factor-at-a-time optimization insufficient for identifying robust operating windows. Consequently, contemporary microneedle development increasingly relies on statistical and computational optimization methodologies capable of evaluating multiple interacting variables simultaneously (Table 13). Design of Experiments (DoE) provides a particularly effective framework because it enables systematic investigation of factor interactions while substantially reducing experimental burden compared with conventional optimization strategies. Response Surface Methodology (RSM) has been successfully applied to optimize geometric parameters and identify optimal operating windows, whereas finite element analysis (FEA) complements experimental optimization by predicting stress distribution, deformation, buckling behavior, and insertion mechanics before prototype fabrication. More recently, integrated DOE–RSM–FEA workflows have demonstrated the ability to simultaneously optimize tip geometry, aspect ratio, and structural robustness while reducing the number of required design iterations, illustrating how multidimensional optimization can accelerate the development of clinically relevant polymeric microneedle systems [146,147].

Taken together, Table 12 and Table 13 illustrate that optimization priorities shift according to therapeutic objectives rather than remaining constant across all microneedle platforms. This observation reinforces one of the central conclusions of the present review: clinically successful microneedles are unlikely to emerge from isolated optimization of polymers, geometry, or fabrication technologies. Instead, translational success requires simultaneous optimization across multiple interconnected variables, where therapeutic objectives define the acceptable operating window throughout development.

Within a function-driven development strategy, these optimization methodologies should not be viewed merely as statistical tools but as mechanisms for translating predefined therapeutic objectives into experimentally validated combinations of material, geometric, fabrication, and application variables that define an acceptable operating window.

### 7.4. Practical Design Principles for Next-Generation Polymeric Microneedles

The development framework proposed in the preceding subsections provides a systematic pathway for selecting and optimizing design variables throughout microneedle development. Collectively, the evidence discussed throughout this review converges into a set of practical design principles that can guide the rational development of next-generation polymeric microneedle systems. These principles do not represent independent recommendations; rather, they summarize the recurring design–performance relationships consistently demonstrated across primary experimental studies and illustrate how interconnected design decisions ultimately determine translational success [6,9,32,40,46,50,52,54,82,93,99].

Principle 1. Begin with the therapeutic objective rather than the material

Successful microneedle development should begin by defining the intended therapeutic outcome before selecting the polymer or fabrication technology. This sequence avoids a common limitation of material-centered development, where promising polymers are optimized extensively before their suitability for the intended pharmacological application has been critically evaluated [93,99].

Principle 2. Optimize the mechanical insertion window instead of maximizing strength

Insertion performance should be evaluated as an emergent property of the complete system rather than as an isolated material characteristic [32,106].

Principle 3. Match release programming to pharmacological requirements

Drug release should be regarded as a programmable design function rather than an intrinsic consequence of polymer selection. Selecting an inappropriate release mechanism may compromise therapeutic performance even when insertion is successful, highlighting the importance of aligning polymer behavior with pharmacological objectives from the earliest stages of development [93,99].

Principle 4. Treat fabrication as an active design variable

Fabrication should not be viewed as a downstream manufacturing step but as an integral determinant of microneedle performance. Contemporary manufacturing strategies—including advanced micromolding, photocurable polymer systems, and high-resolution additive manufacturing—provide opportunities to tailor mechanical modulus, crosslink density, and internal architecture with considerable precision. However, these advantages must be balanced against increased manufacturing complexity, quality-control requirements, and regulatory considerations [99,106]. The optimal fabrication strategy is therefore the one that consistently reproduces the critical quality attributes required for the intended clinical application rather than the one offering the greatest technological sophistication.

Principle 5. Validate performance through progressive translational evaluation

Reliable translation requires complementary evaluation methods that reflect successive stages of product development. Early formulation optimization is best supported by in vitro release testing, whereas permeation studies, ex vivo skin models, and in vivo investigations progressively establish functional delivery performance and pharmacokinetic relevance. Equally important, human-factor considerations—including application force, insertion reproducibility, patch adhesion, and self-administration—should be incorporated before late-stage development, as these variables frequently determine real-world performance despite promising laboratory results.

Principle 6. Optimize interconnected variables within a defined design space

Perhaps the most important lesson emerging from the current evidence is that no single design parameter can be optimized independently of the others. Polymer chemistry, molecular weight, crosslink density, needle geometry, fabrication conditions, drug loading, application parameters, and skin biomechanics collectively define an acceptable operating window for successful drug delivery. Systematic optimization approaches, including Design of Experiments, response surface methodology, finite element analysis, and emerging computational optimization methods, provide increasingly powerful tools for identifying these multidimensional relationships and refining the design space through iterative experimentation. Rather than searching for universally optimal materials or geometries, developers should identify combinations of variables that satisfy the predefined functional requirements of the intended therapeutic application [6,32].

## 8. Conclusions and Outlook

Polymeric microneedles represent one of the most promising strategies for overcoming the limitations of conventional drug delivery routes, offering the potential to combine the efficacy of parenteral administration with the convenience of minimally invasive systems. However, as demonstrated throughout this review, the rapid expansion of research activity in this field has not been matched by a proportional increase in clinically translated systems. This discrepancy reflects a fundamental challenge: the majority of microneedle designs are optimized for technical feasibility rather than for functional and clinical performance.

A central conclusion emerging from this analysis is that microneedle systems cannot be effectively developed through material- or technology-driven approaches alone. Instead, successful translation requires a function-driven design paradigm in which therapeutic objectives, mechanical performance, release kinetics, and material behavior are integrated from the earliest stages of development. Mechanical integrity must be understood as a system-level property arising from the interplay between polymer characteristics, microneedle geometry, array configuration, and application conditions. Similarly, drug release should be regarded as a programmable function aligned with pharmacological intent, rather than a passive consequence of polymer selection.

The analysis of fabrication strategies, material classes, and mechanical behavior further highlights that no single polymer or manufacturing method is universally optimal. Each system involves inherent trade-offs between mechanical strength, drug loading capacity, reproducibility, scalability, and safety. These trade-offs become particularly critical in the context of clinical translation, where factors such as user variability, polymer fate in the skin, and regulatory requirements impose constraints that are often underrepresented in early-stage research.

Importantly, the recurring failure modes identified in this review—mechanical–insertion mismatch, pharmacokinetic–dose limitations, manufacturing constraints, and clinical misalignment—demonstrate that translational barriers are rarely due to isolated technical limitations. Rather, they arise from cumulative inconsistencies between system design and real-world application conditions. Addressing these challenges requires a shift toward integrated, systems-level optimization supported by physiologically relevant testing and rigorous in vitro–in vivo correlation.

Future research should therefore prioritize the development of standardized evaluation frameworks that couple mechanical characterization with validated skin insertion models, as well as the establishment of robust pharmacokinetic and pharmacodynamic endpoints. In parallel, greater attention must be given to long-term safety, particularly with respect to polymer degradation, accumulation, and repeated-use scenarios. Advances in manufacturing technologies and scalable fabrication processes will also be essential for bridging the gap between laboratory-scale prototypes and clinically viable products.

Finally, it is important to recognize that microneedle systems are not universally applicable to all drug delivery challenges. Their successful implementation depends on careful selection of therapeutics that are inherently compatible with transdermal or intradermal delivery. Within these boundaries, however, function-driven design provides a powerful framework for transforming microneedle technologies from experimental constructs into clinically relevant drug delivery platforms.

Ultimately, the future of polymeric microneedles will depend not on how advanced they are, but on how precisely they are designed to meet real clinical needs.

Beyond identifying the principal determinants of microneedle performance, the proposed comparison of development approaches, decision-making algorithm, and design-space optimization framework provides a practical roadmap for translating function-driven design principles into systematic development strategies that better align laboratory optimization with clinical implementation.

## Figures and Tables

**Table 1:** Qualitative comparison of major microneedle types with respect to key performance and translational parameters.

Type	Solid MNs	Coated MNs	Hollow MNs	Dissolving, Biodegradable and Implantable MNs	Hydrogel-Forming MNs
Mechanism [14,15,16,17,18,20,22,23,24,25,26,28,29]	“poke-and-patch”	“coat-and-poke”	“poke and flow”	“poke-and-release”, “poke-and-implant”	“poke-and-permeate”
Insertion and mechanics parameters					
Insertion efficiency [5,7,38]	Very high	High/very high	Moderate	Low/moderate	Low/moderate
Mechanical strength [32,43,49,50]	Very high	High/very high	Moderate	Low	Low/moderate
Microchannel persistence after removal [11,51,52]	Very low	Very low	Moderate	High	Very high
Drug delivery performance parameters					
Drug loading capacity [11,40,41,43,45,48]	Very low	Low	Very high	Moderate	Moderate/High
Drug retention in the skin [11,43,44]	Low	Moderate/high	Moderate	High	Very High
Controlled drug release ability [11,43,44,46]	Very low	Low	Moderate	Moderate/high	Very high
Bioavailability [15,18,22]	Low/moderate	Moderate/high	Very high	High	High
Precise dosing ability [17,18,22,36]	Very low	Moderate	Very high	High	High
Onset of action [15,18,20,22,28]	Slow	Fast/Very fast	Very fast	Fast	Moderate
Delivery period [26,28,44,48,53]	Very short	Short	Short/moderate	Moderate/long	Long/Very long
Translation and usability parameters					
Risk of pain [20,54]	High	High	Moderate	Very low	Very low
Risk of hazardous waste [6,11,18,22,24]	Very high	Very high	Very high/high	Very low	Low
Fabrication complexity [7,36,43,48]	Low	Moderate	Very high	Moderate	High
Drug delivery variability [6,11,31,48,55]	High	Moderate	Low/moderate	Moderate	Low
Self-administration ability [5,10]	Low/moderate	Moderate	Low	Very high	High
Patient compliance [5,56]	Low	Moderate	Moderate	Very high	High/very high

**Table 2:** Fabrication methods for polymeric microneedles: advantages and limitations.

Fabrication Method	Principle	Advantages	Limitations
Micromolding [57,58]	Casting polymer solution/melt into microstructured molds followed by drying or curing	Simple, versatile; compatible with wide range of polymers; allows drug incorporation; low cost	Sensitive to processing parameters; batch variability; limited scalability; potential defects (air bubbles, incomplete filling)
Drawing-based methods [60,63]	Stretching polymer droplets into microneedle structures using controlled airflow or mechanical force	Rapid fabrication; no mold required; sharp tip formation; suitable for prototyping	Poor control over uniformity; limited reproducibility; difficult scale-up; high process variability
3D printing (SLA/DLP) * [12,61]	Layer-by-layer photopolymerization to build microneedle structures	High design flexibility; customizable geometries; rapid prototyping; potential for personalized devices	Limited material selection; insufficient resolution for sharp tips (in some systems); post-processing required
Hot embossing/Injection molding [59]	Thermoplastic polymer shaping under heat and pressure in microstructured molds	High reproducibility; industrial scalability; precise geometry control	High temperature may degrade drugs; limited to thermoplastic polymers; complex equipment
Layer-by-layer/hybrid fabrication [53]	Sequential deposition or combination of multiple fabrication techniques	Enables multilayer structures; spatial drug distribution; advanced functionalities	Increased complexity; reproducibility challenges; difficult regulatory validation

* SLA (Stereolithography) and DLP (Digital Light Processing).

**Table 3:** Major polymer classes used for the fabrication of polymeric microneedles: mechanisms, advantages, limitations, and representative examples.

Water-Soluble Polymers	Biodegradable Polymers	Hydrogel Polymers
Mechanism/function		
Form dissolving MN matrices that rapidly hydrate and dissolve upon contact with interstitial fluid, releasing the incorporated drug into the skin	Form solid or matrix-type MNs that gradually degrade in the skin, allowing prolonged and controlled drug release; degradation occurs through hydrolysis and/or enzymatic processes depending on polymer chemistry	The MN matrix itself generally does not dissolve during application; instead, it forms crosslinked hydrophilic MN networks that absorb interstitial fluid, swell and form hydrated pathways through which drug from a reservoir can diffuse.
Examples		
Poly(vinylpyrrolidone) (PVP); poly(vinyl alcohol) (PVA); hyaluronic acid (HA); carboxymethylcellulose (CMC); maltose; trehalose	Poly(lactic-co-glycolic acid) (PLGA); polylactic acid (PLA); polycaprolactone (PCL); poly(lactic acid) (PLA)-based copolymers	Poly(acrylic acid) (PAA); poly(methyl vinyl ether-co-maleic anhydride) (PMVE/MA); polyethylene glycol (PEG)-based networks; poly(vinyl alcohol) (PVA) hydrogels; hyaluronic acid-based crosslinked systems
Advantages		
Rapid drug release; no residual sharp waste; rapid polymer clearance; generally good biocompatibility; relatively simple processing; suitable for single-step application	Sustained and long-acting drug delivery; tunable degradation and release kinetics; potential for high structural integrity; suitable for poorly soluble drugs and depot-type delivery; degradation products can be eliminated through normal biological pathways	Sustained and controlled drug delivery; high water uptake; MNs remain structurally intact and can be removed after application; minimal polymer deposition in skin; swelling can facilitate prolonged drug transport
Disadvantages		
Relatively limited mechanical strength; risk of deformation or fracture during insertion; dissolution rate can be difficult to control; drug loading may be limited; sensitive to moisture	Slow degradation may result in prolonged polymer residence; acidic degradation products from polyesters may affect drug stability or cause local irritation; release kinetics can be difficult to predict; residual polymer may remain in tissue for extended periods; more complex manufacturing	Drug release is primarily diffusion-controlled; relatively slow onset compared with dissolving MNs; requires adequate patch–skin contact; crosslink density must balance mechanical strength and swelling; high swelling may compromise structural integrity or adhesion
References		
[10,65].	[9,66]	[10,11]

**Table 4:** Systems-level framework of the principal domains and subdomains contributing to translational failure in polymeric microneedles.

**Mechanical–Insertion Mismatch** Failure to achieve reliable skin penetration while maintaining structural integrity. [7,12,58,73,74]							
Microneedle geometry: Shape Length Base diameter Tip diameter, etc.		Polymer mechanical properties: Elastic modulus Fracture strength Flexibility Molecular weight		Skin-related factors: Skin thickness Hydration Elasticity Anatomical site		Application-related factors: Insertion force Application angle Application method Residence time	
**Pharmacokinetic–Dose Mismatch** Failure to achieve the desired drug exposure despite successful insertion. [30,58,75]							
Drug physicochemical properties: Molecular weight Solubility Lipophilicity Ionization (pKa) Stability	Drug loading constraints: Matrix loading capacity Therapeutic dose requirements Drug distribution within the MN		Release behavior: Dissolution rate Hydrogel swelling Polymer degradation		Skin transport limitations: Diffusion through microchannels Skin partitioning Microchannel closure		Systemic pharmacokinetics: Absorption rate Sustained exposure Bioavailability
**Manufacturing and Scalability Limitations** Failure to reproduce laboratory performance under industrial production. [58,74,75]							
Material processing: Polymer viscosity Mold filling Drying conditions Crosslinking	Manufacturing consistency: Dimensional uniformity Needle integrity Drug content uniformity		Scale-up challenges: Batch-to-batch reproducibility Automation compatibility Production throughput		Product stability: Moisture sensitivity Storage stability Packaging requirements		Quality and regulatory considerations: Mechanical quality control Sterility GMP
**Clinical–Context Misalignment** Failure of otherwise functional devices to meet real-world clinical and patient requirements. [58,74,75]							
Patient usability: Self-administration Dexterity Training Compliance	Patch performance during wear: Adhesion Movement Wear time		Clinical application factors: Anatomical site selection Skin preparation Environmental conditions		Safety considerations: Skin irritation Residual polymer Infection risk		Clinical implementation: Target population Therapeutic indication Healthcare workflow Cost-effectiveness

**Table 5:** Qualitative comparison of the effects of conical, pyramidal, beveled, and arrowhead microneedle geometries on key performance characteristics relevant to transdermal drug delivery, including skin penetration, insertion force, mechanical strength, tissue interaction, drug retention, and fabrication complexity. The qualitative rankings represent general trends reported in the literature and may vary depending on material composition, dimensions, and fabrication method.

Performance Characteristics	Conical	Pyramidal	Beveled Tip	Arrowhead
Initial skin puncture [5,32,79]	Moderate/High	High/Very high	High/Very high	Very high
Insertion force [32,71,82]	Low/Moderate	Moderate/High	Low	High
Mechanical strength [49,83]	High	Very high	Low/Moderate	Low/Moderate
Risk of incomplete insertion [80,84]	Low/Moderate	Moderate	Moderate	Low
Bending/buckling risk [80,83]	Moderate/High	Low	High	Low/Moderate
Risk of pain or tissue damage [5,85]	Low	Low/Moderate	Moderate/High	Very high
Risk of accidental removal [71]	High	Moderate	Moderate/High	Low
Drug retention in the skin [82]	Low	Moderate	Moderate	Very high
Fabrication complexity [86]	Low	Moderate	High	Very high
Suitability for polymeric microneedles [11]	Very high	Very high	High	Low

**Table 6:** Influence of microneedle geometry on mechanical performance, supported by representative studies. The qualitative rankings represent general trends reported in the literature and may vary depending on material composition and fabrication method.

	↑ Length	↑ Base Diameter	↑ Aspect Ratio	↑ Tip Diameter/Apex Angle	↑ Interspacing
Drug loading capacity [88,91]	↑	↑			↓
Skin deformation [50,92]	↑	↑			↓
Penetration depth [50,92]	↑	↓	opt. *	↓	
Risk of pain [54]	↑	↑		↑	
Fabrication complexity [49,91]	↑	↑	↑	↑	
Bending/buckling resistance/forces [43,50]	↓	↑	↓	↑	
Mechanical strength [43,49,50]	↓	↑	↓	↑	
Required insertion force [50,55,92]	↑	↑		↑	↓
Bed-of-nail effect [50]		↑			↓
Insertion reliability/ratio [50,55,92]	↑	↓	↑/opt.	↓	↑

* opt.—optimal.

**Table 7:** Influence of polymer physicochemical, mechanical, rheological, surface, and processing properties on the manufacturing quality and drug delivery performance of polymeric microneedle systems.

Factor	Main Influence On	General Effect on Microneedle Performance and Drug Delivery
Molecular weight [7,12,41,56,100,101]	strength; viscosity of casting solution; mold filling ability; dissolution rate; swelling ability; drug diffusion through the matrix.	Higher molecular weight generally increases mechanical strength and viscosity, but slows dissolution and drug diffusion.
Polymer concentration [11,12,73,102]	needle formation; structural integrity (brittleness or flexibility); drug loading capacity; dissolution/swelling kinetics; diffusion through the matrix.	Higher concentration usually improves structural integrity and drug loading, but may reduce dissolution and increase brittleness.
Polymer type [7,12]	mechanical performance; hydrophilicity/hydrophilicity; biocompatibility; biodegradability; drug–polymer interactions; release behavior.	Determines mechanical performance, water uptake, biodegradability, drug interactions, and release behavior.
Hydrophilicity/hydrophobicity [86,103]	swelling; dissolution; drug transport.	More hydrophilic polymers swell or dissolve faster and usually enhance drug release.
Crosslink density [47,86,103]	Mechanical strength; swelling, drug diffusion; release duration.	Higher crosslinking increases mechanical strength but decreases swelling and drug diffusion.
Swelling capacity [47,86,103]	drug diffusion; release duration.	Greater swelling facilitates diffusion and may prolong drug release.
Dissolution rate [11,73,100]	drug release; delivery efficiency.	Faster dissolution accelerates drug release but may shorten delivery duration.
Glass transition temperature (Tg) [7,11,73]	rigidity at room and body temperature; storage stability; needle deformation during insertion; moisture sensitivity.	Higher Tg generally improves rigidity and resistance to deformation during storage and insertion.
Crystallinity [7,100]	strength; dissolution; moisture uptake; drug stability; processability.	Higher crystallinity increases rigidity and stability but slows dissolution and reduces water uptake.
Residual moisture [7,73,104,105]	mechanical strength; stability, insertion; brittleness; tip sharpness; storage stability; premature swelling or dissolution.	Higher moisture content softens the matrix, reduces insertion efficiency, and may compromise stability.
Mechanical modulus [7,12,32,51,106]	buckling; fracture; insertion; elastic modulus; tensile strength; fracture toughness; flexibility.	Higher modulus improves penetration and resistance to bending or fracture.
Rheology [7,12,73,107,108]	mold filling; reproducibility; morphology; air bubble entrapment; tip replication; layer uniformity.	Critical for micromolding and multilayer fabrication.
Film-forming and drying properties [63,73,100]	needle morphology; surface smoothness; crack formation; shrinkage; dose uniformity.	Rapid or uneven drying may cause defects and drug migration.
Drug–polymer interactions [73,100]	loading; stability; release.	Strong interactions may improve drug stability but can retard drug release.
Polymer blend composition [7,73]	strength; dissolution; swelling; release kinetics; processability.	Blending polymers can balance mechanical performance and release characteristics.
Mesh size/pore size [86,103]	diffusion; sustained release.	Larger mesh size enhances drug diffusion, whereas smaller mesh size prolongs release.
Degradation/erosion behavior [5,86]	release duration; biocompatibility; stability during storage; In vivo persistence.	Faster degradation accelerates release, while slower degradation supports prolonged delivery. Important for biodegradable and long-acting systems.
Surface properties of the polymer [5,7,58]	wettability; adhesion to skin; friction during insertion; coating efficiency; protein adsorption.	Influences insertion and biological interactions.
Thermal and processing stability [7,63,73]	sterilization compatibility; drying temperature tolerance; compatibility with APIs; shelf-life.	Essential for industrial manufacturing.

**Table 8:** Skin and application-related factors affecting the performance and drug delivery efficiency of polymeric microneedles.

Factor	Examples	Main Influence On	General Effect on Microneedle Performance and Drug Delivery	Generally Preferable Condition
Application mode [12,115,116,117]	Manual finger or applicator-assisted application; Dynamic impact insertion; Press-and-hold application.	insertion depth; insertion reproducibility; drug delivery variability; patient usability.	Determines the consistency of skin penetration and dose delivery.	Standardized applicator-assisted modes generally improve reproducibility.
Insertion force [12,118]	Magnitude of force; Uniformity of force; Force distribution across the array.	successful skin penetration; microchannel formation; incomplete insertion; delivered dose.	Insufficient or non-uniform force can reduce penetration efficiency and increase variability in drug delivery.	Standardized force sufficient to exceed the insertion threshold and uniformly transmitted across the array
Application duration/residence time [12,119,120]	Short application; Intermediate application; Prolonged application.	polymer dissolution; hydrogel swelling; drug release; delivered dose.	Adequate residence time is required to achieve sufficient polymer dissolution or hydrogel swelling and reproducible drug delivery.	Formulation-specific, standardized residence time sufficient for the intended dissolution/swelling and drug delivery.
Anatomical application site [12,90,118,121,122,123]	Upper arm; Forearm; Abdomen; Thigh; Back; Face.	penetration depth; drug permeation; absorption; local/systemic exposure.	Different skin sites produce different absorption profiles and bioavailability.	Abdomen or upper arm is the most common.
Skin condition before application [123]	Hydrated vs. dry skin; Healthy vs. diseased skin; Hairy vs. non-hairy skin; Intact vs. damaged skin; Removal of excess surface lipids/moisture; Skin temperature.	insertion efficiency; dissolution/swelling rate; drug permeation. irritation risk.	Skin hydration and barrier integrity can substantially affect MN insertion, dissolution/swelling, and drug permeation.	Clean, intact, adequately hydrated skin with a preserved barrier and minimal hair interference.
Patch fixation and adhesion [124,125]	Adhesive strength; Occlusion; Edge lifting; Movement during wear.	skin contact; insertion; stability; sustained delivery, dose; reproducibility.	Insufficient adhesion or patch displacement can reduce effective skin contact and compromise delivery consistency.	Uniform, sufficient adhesion throughout the intended application period.
Application angle [126]	Perpendicular insertion (90°); Oblique insertion.	penetration depth; tissue localization; needle fracture risk; insertion uniformity.	Insertion angle strongly affects penetration depth and delivery localization; the optimal angle depends on the desired skin target and MN design.	Perpendicular application generally provides more uniform insertion and lower fracture risk.
User-related factors [116,127]	Training/experience; Hand strength; dexterity; Application technique; Compliance with instructions.	self-administration success; dose variability; real-world performance.	User-dependent variation in application technique and force can substantially affect insertion efficiency and dose reproducibility.	Trained user or standardized applicator.
Environmental conditions during application [123,125]	Humidity; Ambient temperature; Skin moisture/Sweating.	premature dissolution; adhesion; polymer stability.	Excessive moisture and temperature extremes may alter polymer behavior and compromise patch adhesion or MN performance.	Controlled temperature and low-to-moderate skin moisture.
Post-application handling [116,128]	Application duration; Patch movement; Premature removal; Physical activity.	drug retention; microchannel closure; delivery duration.	Premature removal or excessive movement during wear can reduce effective MN–skin contact and shorten the delivery period.	Maintain stable contact for the intended application period and minimize patch displacement.

**Table 9:** Drug-related physicochemical factors affecting the performance and drug delivery efficiency of polymeric microneedles.

Major Factor	Main Influence on Delivery Efficiency	General Effect on Microneedle Performance and Drug Delivery
Molecular size [12,85]	Diffusion through polymer and skin; permeation rate	Smaller molecules generally diffuse more readily through the polymer matrix and skin
Lipophilicity [12,85,130]	Partitioning into skin; permeation	Moderate lipophilicity is generally favorable for transdermal permeation; very hydrophilic or highly lipophilic compounds may exhibit limited permeation.
Aqueous solubility [85,131]	Drug dissolution, release, and thermodynamic activity	Poorly soluble drugs may show incomplete dissolution and limited delivery.
Ionization (pKa, degree of ionization, pH) [4,130]	Solubility, skin partitioning, and permeation	The non-ionized fraction generally exhibits greater partitioning into the lipid-rich stratum corneum.
Solid-state properties (polymorphism, amorphous/crystalline state, melting point) [130,132]	Drug dissolution, stability, and release	Amorphous drug may provide increased apparent solubility but can present greater physical instability.
Chemical and physical stability [6,85]	Drug integrity during manufacture, storage, and application	Unstable drugs may undergo degradation during formulation, drying, storage, or application, reducing the effective delivered dose and potentially generating degradation products.
Drug–polymer interactions [6,12]	Drug loading, retention, dissolution, and release	Strong interactions may improve drug retention and loading but can slow dissolution/release or cause incomplete drug recovery. Weak or poorly controlled interactions may result in drug migration or crystallization.
Drug–skin interactions (Partition coefficient, binding to skin components) [24,130]	Skin retention and transdermal permeation	Excessive skin binding may increase local retention and reduce systemic availability.
Dose/loading requirement [24,83]	Feasibility of loading and achieving therapeutic delivery	High-dose drugs may exceed the loading capacity of MN arrays and can compromise mechanical strength, needle formation, dissolution, or patch dimensions.
Diffusion coefficient [85,131]	Release driving force and permeation rate	Higher diffusivity generally facilitates faster drug release, whereas strong matrix interactions can reduce diffusivity.
Thermodynamic activity (drug activity; degree of saturation/supersaturation) [130]	Release driving force and permeation rate	Increasing thermodynamic activity can enhance transdermal flux

**Table 10:** Alignment between release strategy, polymer selection, and therapeutic objective.

Therapeutic Goal	Release Profile	Polymers	Ref.
Vaccination	Immediate	PVP, PVA	[40]
Local anesthesia	Rapid	PVP, PVA	[133]
Chronic therapy	Sustained	PLGA	[43]
Long-term delivery	Extended	PCL, PLGA	[99,133]
Biosensing	Diffusion-controlled	Hydrogels	[46]
Smart delivery	Triggered	PNIPAm, hybrids	[134,135]

**Table 11:** Systematic comparison of microneedle development approaches.

Material-Driven Approach	Technology-Driven Approach	Function-Driven Approach
Starting point		
Selection of a promising polymer	Selection of a fabrication platform	Definition of therapeutic objective and target product profile
Primary optimization focus		
Polymer properties, mechanical strength, dissolution	Geometry, manufacturing precision, scalability	Coordinated optimization of polymer, geometry, fabrication, release, and application conditions
Strengths		
Rapid material screening	Manufacturing innovation	Integrated optimization
Limitations		
Late therapeutic alignment	Performance mismatch	Requires iterative optimization across multiple interconnected variables

**Table 12:** Design-space optimization framework for polymeric microneedles.

Development Stage	Primary Design Objective	Critical Design Variables	Critical Quality Attributes	Representative Optimization Methods	Representative Characterization Methods
TPP *	Define therapeutic performance	Dose, onset, duration, target tissue	Clinical suitability	QbD * planning	Clinical requirements
Polymer selection	Achieve required material behavior	Mw, concentration, Tg, degradation	Strength, dissolution	DoE * screening	Compression, DSC *, FTIR *
Geometry optimization	Reliable skin insertion	Length, tip diameter, spacing, aspect ratio	Insertion efficiency	Response surface methodology	Insertion tests, OCT *
Fabrication optimization	Manufacturing reproducibility	Micromolding, SLA, DLP	Dimensional consistency	Process optimization	SEM *
Release programming	Desired pharmacokinetics	Dissolution, swelling, degradation	Release kinetics	Formulation optimization	IVRT *
Skin performance	Functional drug delivery	Force, residence time, anatomical site	Delivered dose	Application optimization	IVPT, ex vivo skin models
Translation	Clinical robustness	Stability, usability, scalability	Manufacturability	Risk assessment	Stability, human factor studies

* TPP—target product profile; SLA—stereolithography; DLP—digital light processing; QbD—quality by design; DoE—design of experiments; DSC—differential scanning calorimetry; FTIR—Fourier-transform infrared spectroscopy; OCT—optical coherence tomography; SEM—scanning electron microscopy; IVRT—in vitro release testing; IVPT—in vitro permeation testing.

**Table 13:** Representative optimization methodologies used for establishing and refining the polymeric microneedle design space.

Optimization Methodology	Purpose	Typical Variables	Representative Application
One-factor-at-a-time	Preliminary screening	Polymer concentration, needle length	Initial formulation selection
Design of Experiments (DoE)	Identify interactions	Needle height, base diameter, polymer composition, process parameters	Screening significant variables
Response Surface Methodology	Optimize continuous variables	Tip angle, aspect ratio, crosslink density	Define optimal operating window
Finite Element Analysis (FEA)	Predict mechanical behavior	Tip radius, insertion force	Mechanical and insertion modeling
DoE–FEA integrated optimization	Multi-objective optimization	Geometry + material properties	Mechanical and insertion optimization
Machine-learning-assisted optimization	Predict multidimensional performance	Combined formulation and geometry datasets	Accelerated design-space exploration

## Data Availability

No new data were created or analyzed in this study. Data sharing is not applicable to this article.

## Supplementary Materials

The following supporting information can be downloaded at: https://www.mdpi.com/article/10.3390/jfb17100512/s1, Figure S1: Comparison of material-driven, technology-driven, and function-driven development workflows for polymeric microneedles; Table S1: Representative experimental examples illustrating function-driven optimization in polymeric microneedle development; Table S2: Literature-derived reference framework for commonly used qualitative descriptors in polymeric microneedle research; Table S3: Representative translational milestones in microneedle technology and their implications for future polymeric microneedle development.

## Abbreviations

The following abbreviations are used in this manuscript:

APIs	Active pharmaceutical ingredients
CMC	Carboxymethylcellulose
CLIP	Continuous liquid interface production
DLP	Digital light processing
DoE	Design of experiments
DSC	Differential scanning calorimetry
FDA	United States Food and Drug Administration
FEA	Finite Element Analysis
FTIR	Fourier-Transform Infrared Spectroscopy
GMP	Good manufacturing practice
GelMA	Gelatin methacryloyl
HA	Hyaluronic acid
HPLC	High-performance liquid chromatography
ISO	International Organization for Standardization
IVRT	In vitro release testing
IVPT	In vitro permeability testing
MN	Microneedle
MNs	Microneedles
NIR	Near-infrared
PAA	Poly(acrylic acid)
PCL	Polycaprolactone
PEGDA	Polyethylene glycol diacrylate
PEG	Polyethylene glycol
PLA	Poly(lactic acid)
PLGA	Poly(lactic-co-glycolic acid)
PMVE/MA	Poly(methyl vinyl ether-co-maleic anhydride)
PNIPAm	Poly(N-isopropylacrylamide)
PVA	Poly(vinyl alcohol)
PVP	Poly(vinylpyrrolidone)
SLA	Stereolithography
SEM	Scanning electron microscopy
TPP	Target product profile
Tg	Glass transition temperature
QbD	Quality by design
