# Supplementary material for: Beyond Material Selection: Function-Driven Design of Polymer-Based Microneedles for Transdermal Drug Delivery
Source: J Funct Biomater. 2026 Oct 11;17(10):512. doi: 10.3390/jfb17100512 (PMC13706377; doi:10.3390/jfb17100512)
Supplement: Supplementary file 1 [file jfb-17-00512-s001.zip › jfb-4568615-supplementary.pdf]

Table S1. Representative experimental examples illustrating function-driven optimization in polymeric microneedle development.

Design challenge	Implemented solution	Experimental evidence	Design implication
Incomplete insertion despite high fracture force	Sharpened tip geometry	Improved skin penetration	Optimize local stress generation rather than bulk strength alone.
Tip deformation	Optimized PVA concentration	Improved integrity	Polymer concentration has an optimum
Rapid burst release	Higher crosslink density	Sustained release	Network architecture programs release
Variable insulin delivery	Standardized application force	Higher insulin permeation	Application conditions should be incorporated into device design
Manufacturing variability	Optimized micromolding	Better dimensional consistency	Process robustness underpins translation.
Variable home-use performance	Applicator-assisted insertion	More reproducible penetration	Human factors are critical design variables.

Table S2. Literature-derived reference framework for commonly used qualitative descriptors in polymeric microneedle research.

**Qualitative descriptor**	**Representative quantitative range***	**Typical measurement**
Short needle	4	Height/base diameter
Minimal deformation	20%	Axial compression test
Reliable insertion	≥80% penetration efficiency	Skin/Parafilm® insertion
High insertion efficiency	≥90% penetration efficiency	Skin/Parafilm® insertion
Adequate fracture resistance	Fracture force exceeding insertion force with appropriate safety margin	Mechanical compression

*Representative literature ranges; these values should be interpreted as guidance rather than universal acceptance criteria because they depend on polymer mechanical properties (elastic modulus, crystallinity, glass transition temperature), needle geometry, fabrication method, and testing conditions.

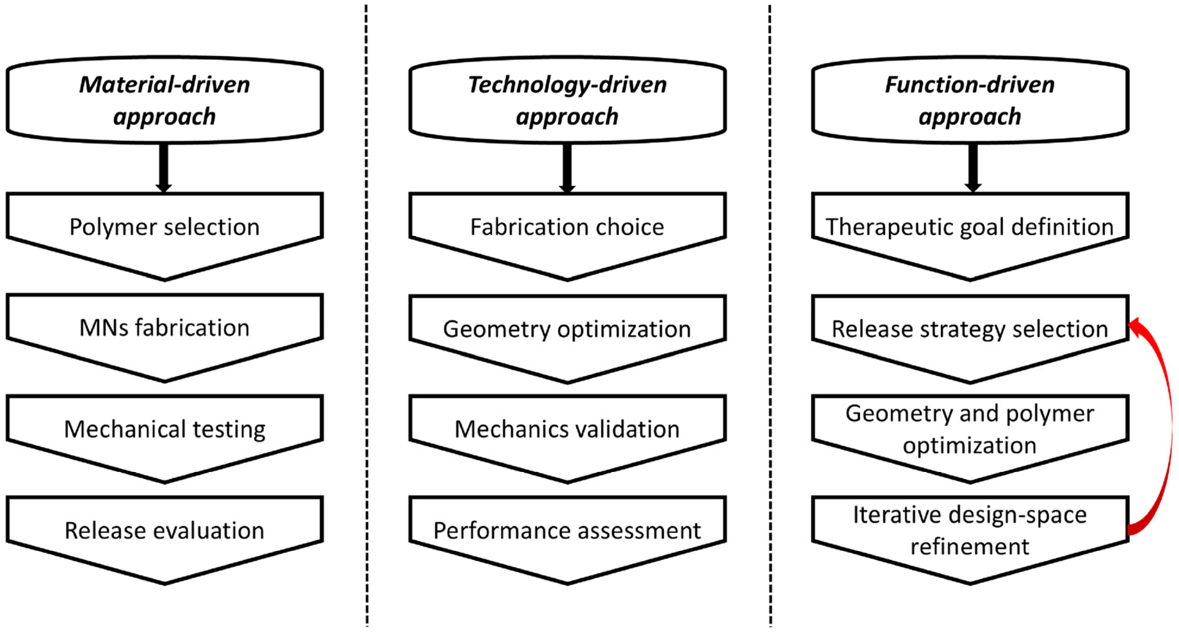

Figure S1. Comparison of material-driven, technology-driven, and function-driven development workflows for polymeric microneedles. Unlike conventional sequential workflows, the proposed function-driven framework integrates therapeutic objectives with iterative optimization of interconnected design variables throughout development.

Table S3. Representative translational milestones in microneedle technology and their implications for future polymeric microneedle development.

System	Highest development stage reached	Platform	Translational insight
MicronJet	Commercial	Hollow microneedles	Standardized applicator-controlled insertion improves dosing reproducibility and clinical usability.
Qtrypta	Late-stage clinical development / FDA review history	Coated microneedles	Manufacturing consistency and regulatory robustness remain critical determinants of commercialization.
MicroCor	Clinical evaluation	Dissolving microneedle patch platform	Wearability, patient acceptance, and practical usability influence long-term adoption.
Dissolving influenza microneedle patches	Phase I clinical evaluation	Dissolving polymeric microneedles	Self-administration is feasible and well accepted, although commercial translation remains under development.
